# CEBPD-mediated SGPP2 upregulation via PERK/ER stress in endothelial cells disrupts S1P homeostasis and impairs angiogenesis in chronic endometritis

**DOI:** 10.1186/s12967-025-07558-0

**Published:** 2025-12-17

**Authors:** Yanjun Wang, Xiaoyan Chen, Guanying You, Shuyi Yu, Cong Chen, Ruochun Lian, Lianghui Diao, Yuye Li, Tailang Yin

**Affiliations:** 1 Reproductive Medicine Center, Renmin Hospital of Wuhan University, No. 99, Zhang Zhidong Road, Wuchang District, Wuhan, Hubei Province 430060 China; 2 Shenzhen Key Laboratory of Reproductive Immunology for Peri- implantation, Shenzhen Zhongshan Institute for Reproductive Medicine and Genetics, Shenzhen Zhongshan Obstetrics & Gynecology Hospital (formerly Shenzhen Zhongshan Urology Hospital), No. 1001, Fuqiang Road, Futian District, Shenzhen, Guangdong Province 518045 China; 3 Guangdong Engineering Technology Research Center of Reproductive Immunology for Peri-implantation, Shenzhen, China

**Keywords:** Chronic endometritis, Sphingosine-1-phosphate (S1P), Angiogenesis, SGPP2, Endoplasmic reticulum stress

## Abstract

**Background:**

Chronic endometritis (CE) is a persistent inflammatory condition associated with adverse pregnancy outcomes. Although impaired endometrial angiogenesis is thought to contribute to its pathogenesis, the underlying molecular mechanisms remain incompletely understood. This study aimed to investigate whether sphingolipid metabolism plays a role in the vascular dysfunction of CE patients.

**Methods:**

Endometrial samples from control and CE patients were assessed for angiogenesis using immunohistochemistry. Sequencing data of endometrial tissues indicated dysregulation of sphingolipid metabolism in CE patients. ELISA revealed decreased levels of sphingosine-1-phosphate (S1P) in the endometrium of CE patients and in lipopolysaccharide (LPS)-treated human umbilical vein endothelial cells (HUVECs). Functional assays including tube formation, wound healing, and transwell invasion were performed to evaluate the effects of LPS and S1P on HUVECs. Western blotting was used to explore the signaling pathways through which S1P influences HUVECs function after LPS stimulation. RT-qPCR and Western blot analyses further suggested that the reduction in S1P under inflammatory conditions may be attributable to upregulation of sphingosine-1-phosphate phosphatase 2 (SGPP2). Chromatin immunoprecipitation (ChIP) and dual-luciferase reporter assays were employed to detect CEBPD binding to the SGPP2 promoter, and immunofluorescence was used to assess nuclear localization of relevant factors. Knockout experiments were conducted to validate the relationship among CEBPD, SGPP2, and endoplasmic reticulum (ER) stress. Finally, the effect of S1P on pregnancy outcomes was evaluated in a CE mouse model.

**Results:**

Microvessel density (MVD) and S1P levels were decreased in both CE patients and the CE mouse model. In HUVECs, LPS suppressed tube formation, migration, and invasion; these effects were reversed by exogenous S1P via the S1PR1-STAT3-VEGFA pathway. SGPP2, an S1P-degrading enzyme, was upregulated in CE endometrial tissues and in LPS-stimulated HUVECs. Mechanistically, the transcription factor CEBPD was shown to directly bind the SGPP2 promoter and promote its expression, a process dependent on PERK-eIF2α-mediated ER stress. In the mouse model, intrauterine administration of S1P attenuated endometrial inflammation, improved angiogenesis, and significantly reduced embryo resorption rates.

**Conclusions:**

Our findings delineate a novel pathway linking inflammatory stress to aberrant angiogenesis in endometrium, in which ER stress-driven CEBPD activation transcriptionally upregulates SGPP2, creating a molecular nexus between inflammation and sphingolipid metabolism. This S1P signaling deficit compromises a critical angiogenic pathway necessary for vascular remodeling, which in turn disrupts endometrial receptivity and contributes to CE-associated reproductive failures.

**Supplementary Information:**

The online version contains supplementary material available at 10.1186/s12967-025-07558-0.

## Introduction

Chronic endometritis (CE) is a persistent inflammatory disorder of the endometrium, histologically characterized by abnormal plasma cell infiltration within the stroma [1]. CE is often associated with microbial infection, with common pathogens including *Streptococcus*, *Escherichia coli*, *Chlamydia* et al. Intrauterine device or structural abnormalities of the endometrium (such as submucosal fibroids or polyps) may also be contributing factors [2, 3]. Although most patients are asymptomatic or present with mild symptoms, CE is strongly associated with unexplained infertility, recurrent pregnancy loss (RPL), and adverse pregnancy outcomes associated with assisted reproductive technologies (ART) [4]. The prevalence of CE in patients with recurrent implantation failure (RIF) is 30–57% and 10–50% in those with RPL [5, 6]. However, the mechanisms by which CE impairs embryo implantation and pregnancy remain unclear.

Successful embryo implantation, placental development, and pregnancy establishment critically depend on synchronized vascular remodeling and angiogenesis throughout the endometrium [7]. The normal endometrial vascular system provides oxygen, nutrients, and signaling molecules to the embryo, which plays a crucial role in embryo implantation and early placental formation [8]. A Previous report reveals that vascular fragility caused by microerosions or microvascular structural abnormalities in CE frequently results in abnormal uterine bleeding (AUB) [9]. Moreover, there is a significant association between endometrial inflammation and vascular changes [10]. These findings suggest that angiogenic impairment may represent a pathological mechanism linking CE to adverse pregnancy outcomes.

In this study, we performed RNA sequencing analysis on the endometrial tissues of CE patients and found that sphingolipid metabolism was significantly downregulated. Among the molecular pathways implicated in vascular dysfunction, sphingolipid metabolism is emerging as a critical yet underexplored regulator [11]. Sphingosine-1-phosphate (S1P), a biologically active sphingolipid metabolite, plays a central role in angiogenesis, maintenance of the endothelial barrier, and immune cell migration by binding to sphingosine-1-phosphate receptor (S1PR) including S1PR1-S1PR5 [12–14]. Among these, S1PR1 plays a particularly crucial role in vascular function protection and anti-inflammatory effects [15, 16]. The intracellular levels of S1P are tightly regulated by opposing enzymatic activities: sphingosine kinases (SPHKs) mediate its synthesis, whereas sphingosine-1-phosphate phosphatase (including SGPP1 and SGPP2) catalyze its dephosphorylation to generate sphingosine [17, 18]. Our preliminary data showed a significant upregulation of SGPP2 expression in vascular endothelial cells of CE patients. We therefore hypothesize that chronic inflammation in CE induces reprogramming of sphingolipid metabolism, primarily through dysregulation of SGPP2 levels, leading to impaired angiogenesis and compromised pregnancy outcomes. However, the upstream molecular mechanisms underlying this abnormal sphingolipid metabolism require further investigation.

The endoplasmic reticulum (ER) is an essential organelle responsible for protein folding, post-translational modification, and trafficking, as well as the ubiquitination and degradation of misfolded proteins. Disruption of ER homeostasis leads to the accumulation of unfolded or misfolded proteins, triggering a cellular stress response known as ER stress [19]. ER stress is commonly induced by various pathological stimuli, including inflammatory cytokines, which activate the unfolded protein response (UPR) as an adaptive signaling cascade [20]. The ER stress sensors activating transcription factor 6 (ATF6), inositol-requiring enzyme 1 (RE1) and protein kinase R-like endoplasmic reticulum kinase (PERK) trigger transcriptional reprogramming mediated by ATF6-N, XBP1S and ATF4, respectively, to regulate the stress response pathways [21]. Emerging evidence has linked aberrant ER stress to impaired placental development and adverse pregnancy outcomes [22, 23]. Furthermore, ER stress has been shown to modulate endothelial cell survival, proliferation, and angiogenesis primarily through UPR-mediated signaling pathways [24]. The ER serves as a primary site of sphingolipid biosynthesis. Previous studies have shown that ER stress can also increase S1P levels *via* the regulation of SPHK enzymes [25], such as SPHK2 [26]. Therefore, in this study, we aim to investigate the mechanistic link between ER stress and sphingolipid metabolism in the pathogenesis of angiogenic dysfunction in CE.

## Materials and methods

### Patients and tissue samples

Human endometrial tissue was provided by Shenzhen Zhongshan Obstetrics & Gynecology Hospital. Samples were strictly excluded under the following conditions: genetic abnormalities, uterine malformations, endometriosis, antibiotic or hormonal medication use within three months prior to sampling, and other major systemic diseases. Based on our previous report, a case was defined as CE if at least one of 30 randomly selected HPFs showed five or more CD138⁺ plasma cells in the endometrial stroma [27]. All human samples used in this study were reviewed and approved by the Ethics Committee of Shenzhen Zhongshan Obstetrics & Gynecology Hospital (Approval No. SZZSECHU-2024065).

### Immunohistochemistry and tissue immunofluorescence

The samples were preserved in a 4% paraformaldehyde solution and underwent gradual dehydration before being embedded in paraffin. They were then sliced into 4-micrometer thick sections. The sections were subjected to hematoxylin and eosin staining (H&E) staining using standard protocols. For immunohistochemistry, endometrial tissues were stained with the antibodies listed in Supplementary material 1. All immunohistochemistry and tissue immunofluorescence staining was performed on a Leica Bond III Automated Immunostainer (Leica Microsystems, Wetzlar, Germany) using BOND Polymer Refine Detection. The Olympus SLIDEVIEW™ VS200 (VS200; Olympus Corporation, Tokyo, Japan) was used to scan panoramic sections at 400× magnification. Quantitative analysis was performed using the HALO Image Analysis System (Indica Labs, Albuquerque NM, USA) under the supervision of at least one pathologist.

### Cell culture

Human umbilical vein endothelial cells (HUVECs) were purchased from the China Type Culture Collection (GDC0635). HUVECs were cultured in endothelial cells medium (ECM) (Cat# 1001,Sciencell) with 5% fetal calf serum (FBS), 1% endothelial cells growth supplement (ECGS), 1% penicillin and streptomycin at 37 °C in a 5% CO₂ incubator. HUVECs from the 3rd to 8th generation were used for independent replicate experiments.

### Cell transfection

S1PR1 siRNA, CEBPD siRNA, SGPP2 siRNA, PERK siRNA, ATF4 siRNA and control siRNA were purchased from Sangon Biotech. SGPP2 overexpression plasmids and empty vector plasmids were synthesized and designed by GENE CREATE (Jinkairui, Wuhan, China). HUVECs reached 60–80% confluence, Lipofectamine™ 3000 (Invitrogen) was used for cell transfection. First, the siRNA or plasmid and Lipofectamine 3000 were separately diluted in Opti-MEM™ (Thermo Fisher) and incubated at room temperature for 5 min. Next, the siRNA or plasmid was mixed with Lipofectamine 3000 and incubated at room temperature for 15 min. Finally, the mixture was added to the HUVECs culture medium and incubated at 37 °C for 6–8 h. After a further 24 h incubation at 37 °C in 10% FBS medium, transfection efficiency was assessed via qPCR, and proteins were collected for Western blot analysis. HUVECs migration, invasion, and tube formation were evaluated 24 h post-transfection.

### Transwell invasion assay

Transwell invasion assay was performed with 8 μm pores (LABSELECT) and 24-well plates. A total of 2 × 10^4^ cells transfected as indicated were seeded in the upper chamber, and 10% FBS-containing ECM was placed in the lower chamber. The invaded cells in the lower chamber were fixed with 4% paraformaldehyde and stained with 0.1% crystal violet following a 24 h incubation. The number of invasion cells were counted using a microscope.

### Wound healing assay

Cells were plated in 12-well plates and transfected as indicated. Wounds were made by scratching a line using a 200 µl pipette tip. The detached cells were removed by gently washing with PBS. The scraped lines were photographed using an electron microscope immediately after scratching (0 h), 6, 12 and 24 h later. The wound width was measured using ImageJ software, and the percentage of wound healing was calculated as: (initial wound width - wound width at 24 h) / initial wound width × 100%.

## Tube formation assay

The tube formation assay was used to detect the ability of endothelial cells to generate vessels. Matrigel (Corning) and ECM were mixed at a ratio of 1:1. 2 × 10^4^/well HUVECs per well from each group were seeded on the top of 50 µl Matrigel in a 96-well plate and incubated at 37 °C for 6 h. Representative photos of the tube structure were taken using an inverted microscope. The number of tube branch nodes was analyzed using ImageJ software.

### ELISA assay

According to the manufacturer’s instructions, S1P and Sphingosine (SPH) levels were measured in human or mouse endometrial tissues and HUVECs using ELISA kits. The S1P ELISA kit and the SPH ELISA kit were purchased from mibio.

### Cell immunofluorescence

After treating HUVECs with 1 µg/ml LPS for 24 h, cells were fixed with 4% PFA, permeabilized, and washed with PBS. Subsequently, HUVECs were blocked with 3% BSA. Cells were then incubated with primary antibody overnight, followed by incubation with the corresponding FITC-labeled secondary antibody at room temperature for 1 h. Cell nuclei were stained with DAPI. Imaging was performed using High Content Imaging System (PerkinElmer).

### Quantitative PCR analysis (RT-qPCR)

Total RNA was extracted from all samples using the SteadyPure Universal RNA Extraction Kit (Accurate Biology). Reverse transcription was performed using the Evo M-MLV Reverse Transcription Premix Kit with gDNA Removal Reagent (Accurate Biology). RT-qPCR was performed using the SYBR^®^ Green Pro Taq HS Pre-mix qPCR Kit (Accurate Biology). All experiments were conducted according to the manufacturer’s instructions. The mRNA level of the gene of interest was normalized to that of B2M. Primers used were listed in Supplementary material 2.

### Western blot

After rinsing in PBS, all samples were lysed using RIPA lysis buffer (Beyotime) containing 1 mM PMSF, protease inhibitor cocktail, and phosphatase inhibitor cocktail (Servicebio) for 15 min on ice, sonicated for 1 min. Proteins were separated by SDS‒PAGE (Affinibody) and transferred to PVDF membranes (Servicebio). After blocking in Minute Block buffer (Affinibody) for 30 min, the membranes were incubated overnight with the indicated primary antibodies: ATF4 (1:1000,10835-1-AP, Proteintech), PERK(1:1000,24390-1-AP, Proteintech), eIF2α(1:1000,11170-1-AP, Proteintech), Phospho-eIF2α(1:1000,28740-1-AP, Proteintech), VEGF-A(1:3000,ab51745,abcam), VEGFR-2(1:1000,9698 S, CST), SPHK1(1:1000,10670-1-AP, Proteintech), S1PR1(1:1000,55133-1-AP, Proteintech), Vinculin(1:5000,13901T, CST), α-Tubulin(1:3000,#12152,CST), STAT3(1:1000,10253-2-AP, Proteintech), Phospho-STAT3(1:1000,ET1607-39,HUABIO), CEBPD(1:1000,ab245214,abcam), SGPP2(1:500,PA5-42767,Invitrogen), SPNS2(1:2000,DF15844,affbiotech), SGPL1(1:3000,A26855P, ABclonal), SMAD3(1:1000,66516-1-Ig, Proteintech), NF-κB(1:1000,10745-1-AP, Proteintech) in Tris-buffered saline (TBS) supplemented with 0.1% Tween 20 at 4 °C. The membranes were then incubated with horseradish peroxidase (HRP)-labeled IgG secondary antibodies, developed with ECL Chemiluminescence Detection Kit (Biosharp) on an Invitrogen iBright FL1500 (Invitrogen). Band intensities were quantified using ImageJ software.

### Chromatin Immunoprecipitation (ChIP)

The cells were crosslinked with 1% formaldehyde (final concentration) for 10 min by inverting flasks at room temperature and quenched with 0.125 M Glycine for 5 min. The cell pellets were washed repeatedly in PBS and then stored at -80°C. The pellets were lysed in lysis buffer (50 mM HEPES, 150 mM NaCl, 1 mM EDTA, 0.1% SDS, 0.1% sodium deoxycholate, 1% Triton X-100, and complemented with protease inhibitor cocktail) for 10 min. After centrifugation, the supernatant was discarded and the pellet was lysed in lysis buffer and subjected to sonication. Sheared chromatin was incubated with primary antibody bound to the PierceTM Protein A/G Agarose Beads (Thermo Fisher Scientific, Inc.) overnight, followed by elution and reverse cross-linking at 65°C overnight. TE buffer (10mMTris-Hcl, 1mM EDTA) was added to DNA elution buffer, followed by RNase treatment (0.5 mg/mL) at 37°C for 30 min and proteinase K treatment (0.3 mg/mL) at 51°C for 1 h. The DNA was the isolated, purified and amplified by PCR. The SGPP2 promoter primers were as follows: Primer 1: forward (5’-CCTGTCCCCAGTCCT-3’), reverse (5’-GTCCGAACACCCTTG-3’); Primer 2: forward (5’-TGTTTTGCTTTGCCTAT-3’), reverse (5’-GGGACCTGTTCTGCTT-3’); Primer 3: forward (5’-TGGTGGAGCAATAAGA-3’), reverse (5’-GAGACCCCAAAATAGAG-3’).

### Dual luciferase reporter activity assay

The wild-type (WT) and mutant (MT) sequences of the artificially synthesized SGPP2 gene were cloned into the pGL3-basic reporter vector, while the coding sequence (CDS) of the CEBPD gene was cloned into the pcDNA3.1 expression vector. All constructed vectors were validated by sequencing. The pGL3-basic vector was used to express firefly luciferase, while the pRL-TK vector was used to express Renilla luciferase, serving as an internal control. Following cell transfection, the firefly luciferase activity was measured using the Dual-Luciferase^®^ Reporter Assay System (Promega).

### RNA sequencing (RNA-seq)

Total RNA was extracted from human endometrial tissue using Trizol reagent (Invitrogen). Library construction, sequencing, and bioinformatic analysis were performed by Novogene.

### Animals and experimental protocol

Seven-week-old female C57BL/6 mice were housed in a specific pathogen-free (SPF) environment under standard environmental conditions (22–24 °C and 60–70% relative humidity). Mice were randomly divided into three groups. Endometritis was induced by injecting 20 µL of 25 mg/mL LPS (Beyotime) dissolved in PBS into each uterine side using a mouse uterine perfusion device [28, 29]. In the control group, 20 µL of PBS was perfused into each uterine side. In the rescue group, a mixture of 2.5 mg/mL LPS and 0.016 mg/mL S1P (TargetMol) was perfused. Reagent concentrations and durations were determined based on literature and preliminary experiments. On day 7 post-treatment, mice were euthanized by cervical dislocation, followed by abdominal incision and uterine extraction for subsequent experiments. Alternatively, female mice were mated with male mice at a 2:1 ratio 7 days post-treatment. Embryo day 0.5 (E0.5) was defined as the day visible plugs were observed. Mice were euthanized at E13.5. The embryo resorption rate per mouse was calculated to assess pregnancy outcome, defined as resorbed embryos / (resorbed embryos + live fetuses) × 100%.

### Statistical analysis

Statistical analyses were performed using GraphPad Prism 10. Data were presented as mean ± standard deviation (SD). Two groups were compared using t-tests, while one-way ANOVA was used to compare multiple groups. *p* < 0.05 indicated statistical significance.

## Results

### Angiogenesis is impaired in CE patients

**Fig. 1:**
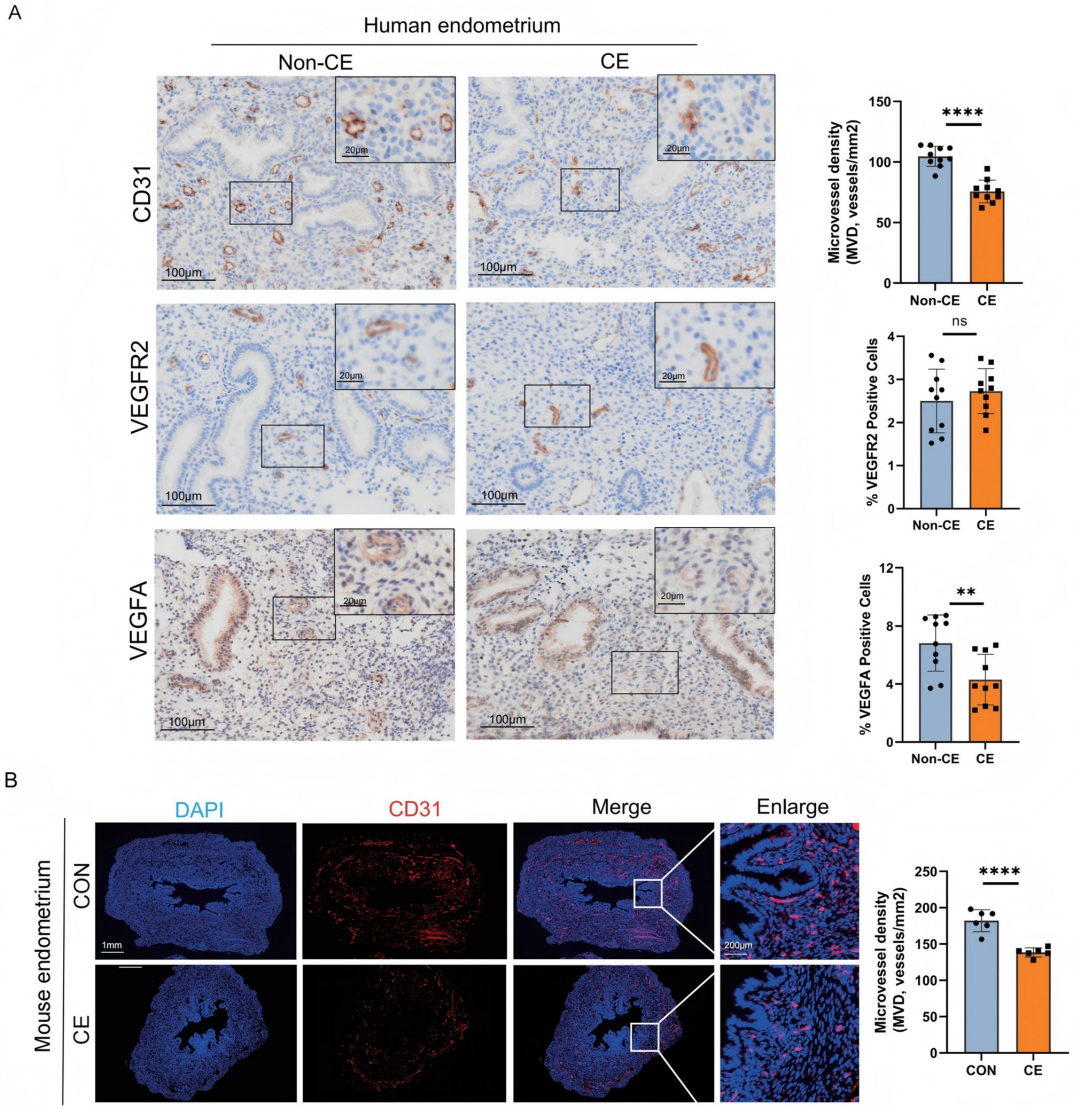
Vascular changes in CE and non-CE patients. (**A**) Immunohistochemical analysis of CD31, VEGFR2, and VEGFA expression in endometrial tissues from CE (*n* = 10) and non-CE patients (*n* = 10). (**B**) Immunofluorescence detection of CD31 in the control (CON) group (*n* = 6) and mouse CE model group (*n* = 6). The HALO Image Analysis System was used to quantitatively analyze the percentage of positive cells and MVD. All data are presented as mean ± SD. *P < 0.05, **P < 0.01, ***P < 0.001, ****P < 0.0001; ns, no significant difference. MVD: Microvessel density. CE: Chronic endometritis

 To further investigate the impact of CE on angiogenesis, we collected endometrial tissue samples from CE patients and mouse endometritis models.Immunohistochemical staining for the angiogenesis markers CD31, VEGFA, VEGFR2 was performed. Statistical analysis showed that MVD and VEGFA expression were significantly decreased in CE patients (Fig. 1A). A mouse endometritis model was established using LPS, and MVD was assessed using CD31 tissue immunofluorescence staining. Consistent with the results in CE patients, MVD in uterine tissue of LPS-treated mice was significantly lower than that in the control group (Fig. 1B).

### CE impairs angiogenesis by affecting S1P production

**Fig. 2:**
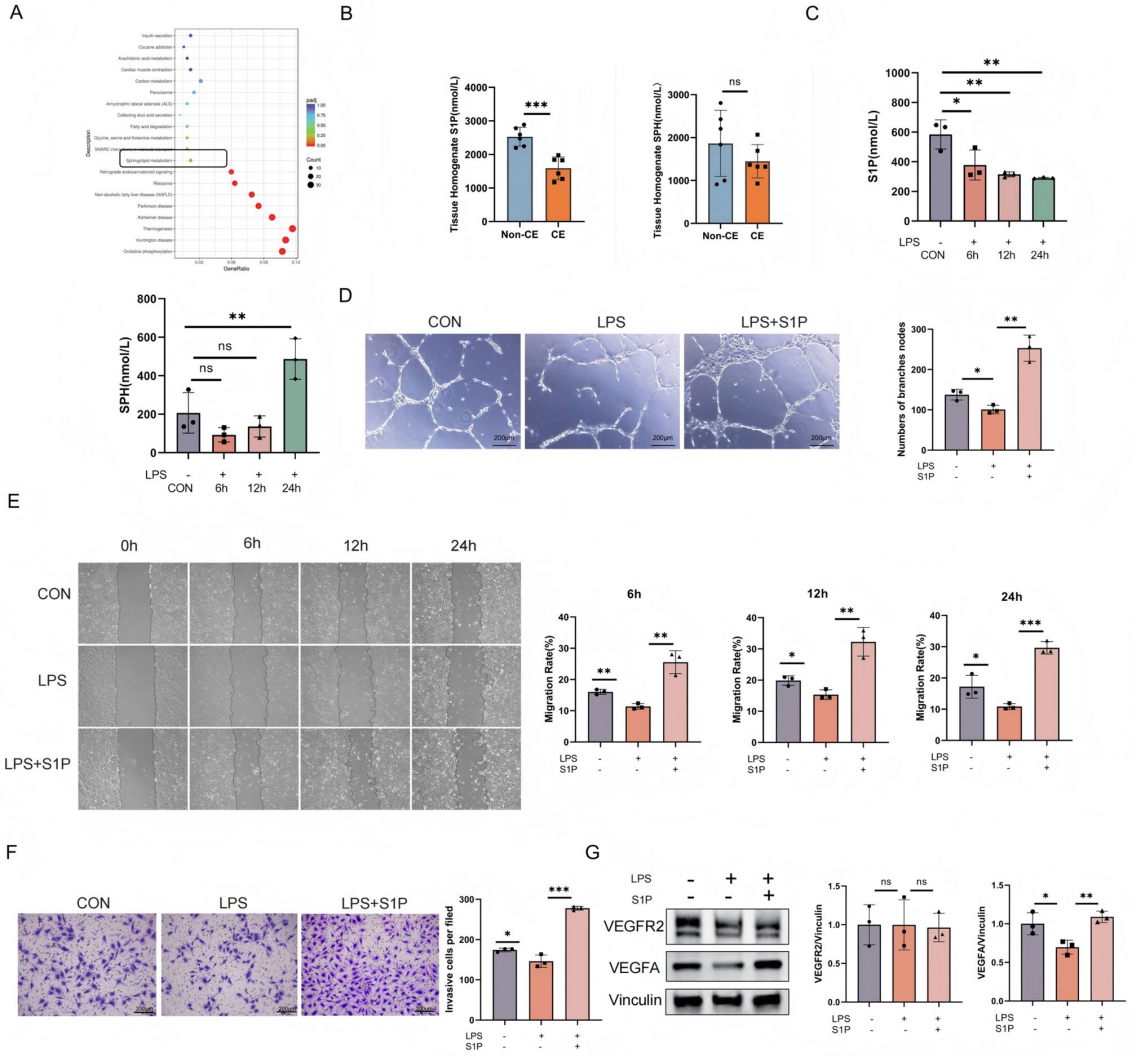
Impaired angiogenesis in CE patients is associated with abnormal S1P metabolism. (**A**) KEGG pathway enrichment analysis of endometrial tissues from CE (*n* = 6) and non-CE (*n* = 6) patients, presented as a bubble plot visualizing significantly enriched signaling pathways (adjusted *P* < 0.05). Pathway relevance is indicated by bubble size (gene count) and Y-axis position (enrichment significance). (**B**) ELISA quantification of S1P and SPH levels in CE and non-CE endometrial tissues (*n* = 6 per group). (**C**) ELISA analysis of S1P and SPH levels in HUVECs stimulated with LPS (1 µg/ml) at 6 h, 12 h, and 24 h. (**D**) Tube formation assay demonstrating rescue of LPS-suppressed angiogenesis by exogenous S1P (0.5 µmol). Branch node counts (quantified via ImageJ) reflect endothelial network formation capacity. (**E**) Wound healing assay tracking LPS- or LPS + S1P-treated HUVECs migration: Images captured at 0 (baseline), 6, 12, and 24 h post-wounding; relative migration rates calculated by ImageJ-based wound area reduction analysis. (**F**) Transwell invasion assay evaluating HUVECs invasiveness (24 h treatment: CON, LPS, or LPS + S1P). Cells suspended in serum-free medium were seeded onto matrix gel-coated chambers; invasive cells on the basolateral surface were counted (3 random fields/view, light microscopy) and quantified via ImageJ. (**G**) Western blot validation of VEGFA and VEGFR2 protein expression in HUVECs (24 h treatment: CON, LPS, or LPS + S1P). Band intensities normalized to loading controls and quantified using ImageJ. All data are presented as mean ± SD. *P < 0.05, **P < 0.01, ***P < 0.001; ns, no significant difference. CON: control

To further investigate the molecular mechanism of CE affecting endometrial angiogenesis, we conducted an RNA sequencing (RNA-Seq) analysis of the endometrial tissue from CE patients and non-CE group. KEGG pathway enrichment revealed significant dysregulation of sphingolipid metabolism (Fig. 2A). S1P is a highly bioactive sphingolipid signaling molecule, generated by the phosphorylation of sphingosine (SPH). It plays a critical role in regulating a wide range of physiological and pathological processes, including cell proliferation, migration, survival, angiogenesis, and immune responses [30]. ELISA analysis further showed that S1P levels were lower in CE patient tissues compared to non-CE patients, while SPH levels exhibited no statistically significant difference (Fig. 2B). We treated HUVECs with LPS to establish an in vitro inflammatory model (Figure S1A). ELISA analysis showed that S1P levels were significantly decreased and SPH levels were significantly increased in HUVECs after LPS treatment (Fig. 2C). Collectively, impaired microvascular density in CE patients may be associated with abnormal sphingolipid metabolism.

We found that LPS treatment significantly inhibited the lumen formation of HUVECs, while supplementing S1P could effectively reverse this effect (Fig. 2D). Wound healing and transwell experiments further indicated that LPS reduced the migration and invasion ability of HUVECs, while supplementing S1P could restore these functions (Fig. 2E, F). AS one of vascular endothelial growth factors, VEGFA plays a central role in the process of angiogenesis. When tissues are stimulated by inflammation, cells release VEGFA, which diffuses into nearby blood vessels and binds to VEGFR2 on the surface of vascular endothelial cells, initiating downstream signaling pathways [31, 32]. As shown in Fig. 2G, VEGFA expression in LPS-treated HUVECs was significantly downregulated, while VEGFR2 expression showed no change. Furthermore, supplementing S1P could reverse the downregulation of VEGFA, suggesting that S1P promotes angiogenesis by maintaining the level of VEGFA rather than affecting VEGFR2 expression (Fig. 2G).

### S1P promotes angiogenesis via the S1PR1- STAT3-VEGFA signaling pathway

**Fig. 3:**
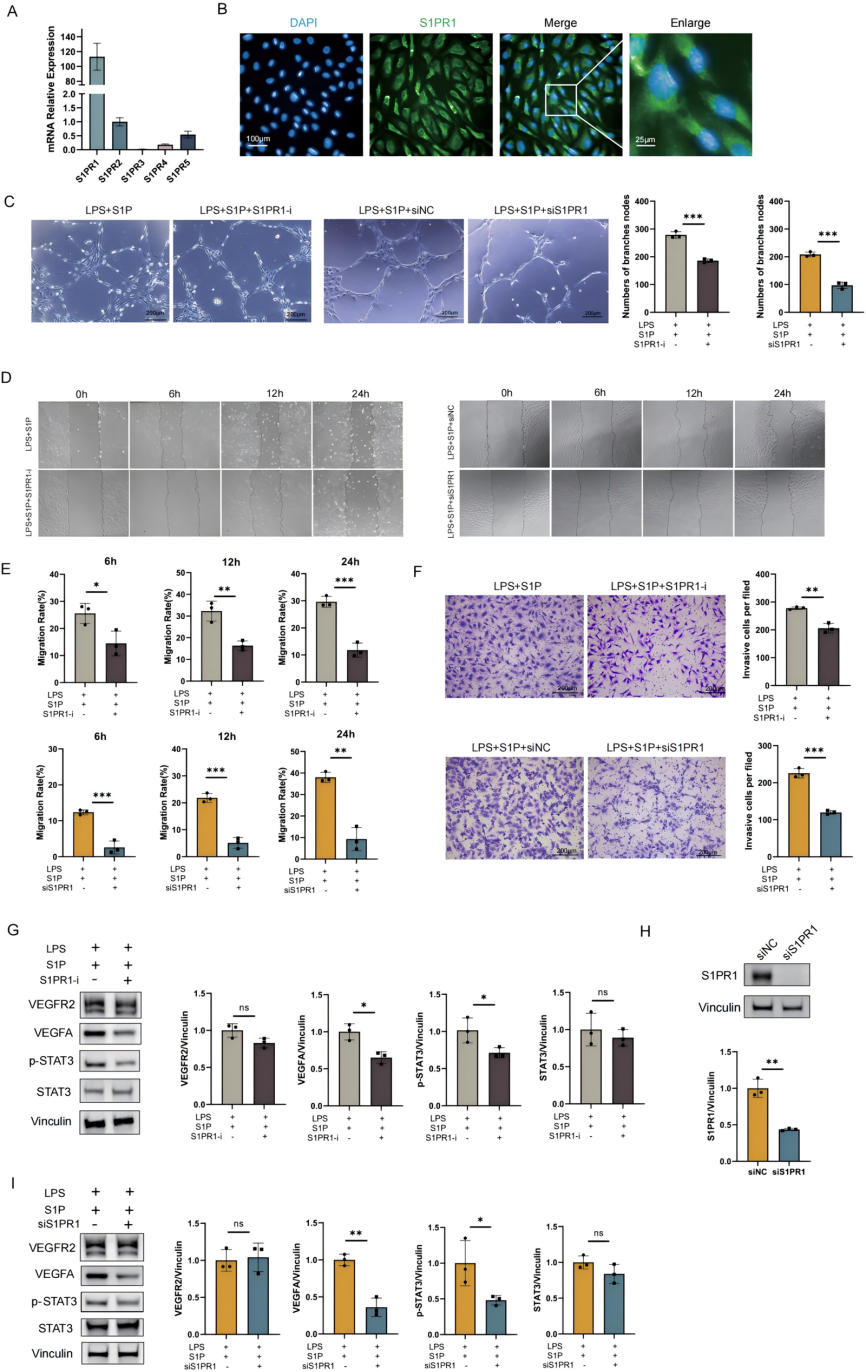
S1P exerts function via the S1PR1-STAT3/-VEGFA pathway. (**A**) qPCR detection of relative mRNA expression levels of S1PR1-5 in HUVECs. (**B**) Immunofluorescence staining of S1PR1 expression in HUVECs, (S1PR1: green, DAPI: blue). (**C**) Tube formation assay assessing the impact of S1PR1 inhibition on LPS + S1P-restored angiogenesis: HUVECs treated with S1PR1 inhibitor W146 (10 µM, S1PR1-i) or S1PR1-targeting siRNA (siS1PR1) during LPS + S1P stimulation. Branch node counts (ImageJ-quantified) reflect endothelial network formation capacity. (**D**, **E**) Wound healing assays evaluating migration under S1PR1 inhibition: Time-lapse imaging (0 h, 6 h, 12 h, 24 h) of HUVECs treated with S1PR1-i or siS1PR1 under LPS + S1P conditions; relative migration rates calculated from wound area reduction (ImageJ). (**F**) Transwell invasion assay quantifying invasive capacity: HUVECs (LPS + S1P + S1PR1-i or LPS + S1P + siS1PR1) seeded onto matrix gel-coated chimeric chambers (serum-free medium). After 24 h, invasive cells on the basolateral membrane surface were counted and quantified via ImageJ. (**G**) Western blot validation of STAT3, phosphorylated STAT3 (p-STAT3), VEGFA, and VEGFR2 expression in HUVECs treated with S1PR1-i under LPS + S1P conditions (vs. untreated controls); band intensities quantified via ImageJ. (**H**) Efficiency verification of siS1PR1 via Western blot (targeting S1PR1 knockdown) and ImageJ-based quantification. (**I**) Western blot analysis of STAT3, p-STAT3, VEGFA, and VEGFR2 in HUVECs treated with siS1PR1 under LPS + S1P conditions (vs. scrambled siRNA controls); band intensities quantified via ImageJ. All data are presented as mean ± SD. *P < 0.05, **P < 0.01, ***P < 0.001; ns, no significant difference

As a key lipid molecule, S1P activates downstream signaling pathways through its receptors S1PR1-5 [13]. RT-qPCR analysis revealed the highest expression of S1PR1 in HUVECs, confirmed by immunofluorescence localization (Fig. 3A, B). To determine whether S1P regulates angiogenesis specifically via S1PR1, we treated HUVECs with the S1PR1 inhibitor W146 or *S1PR1*-targeted siRNA. Notably, inhibition of S1PR1 or S1PR1 siRNA significantly suppressed S1P-mediated rescue of tube formation in LPS-stimulated HUVECs (Fig. 3C). Moreover, inhibition of S1PR1 or S1PR1 siRNA also significantly attenuated S1P-promoted cell migration and invasion (Fig. 3D-F). Given that signal transducer and activator of transcription (STAT3) directly regulates VEGFA expression to control angiogenesis, cell growth, and metastasis [33], we further investigated whether the S1P/S1PR1 axis modulates VEGFA expression through the STAT3 pathway. Western blot analysis revealed that, compared with the control group, S1P failed to induce STAT3 phosphorylation or upregulate VEGFA expression in either the S1PR1 inhibition or S1PR1 siRNA treatment groups (Fig. 3G-I). Finally, these findings suggest that inflammation may disrupt STAT3-related signaling downstream of S1P by targeting the S1P-S1PR1 pathway, ultimately leading to suppressed VEGFA expression.

### Inflammation-induced SGPP2 upregulation in HUVECs drives S1P degradation

**Fig. 4:**
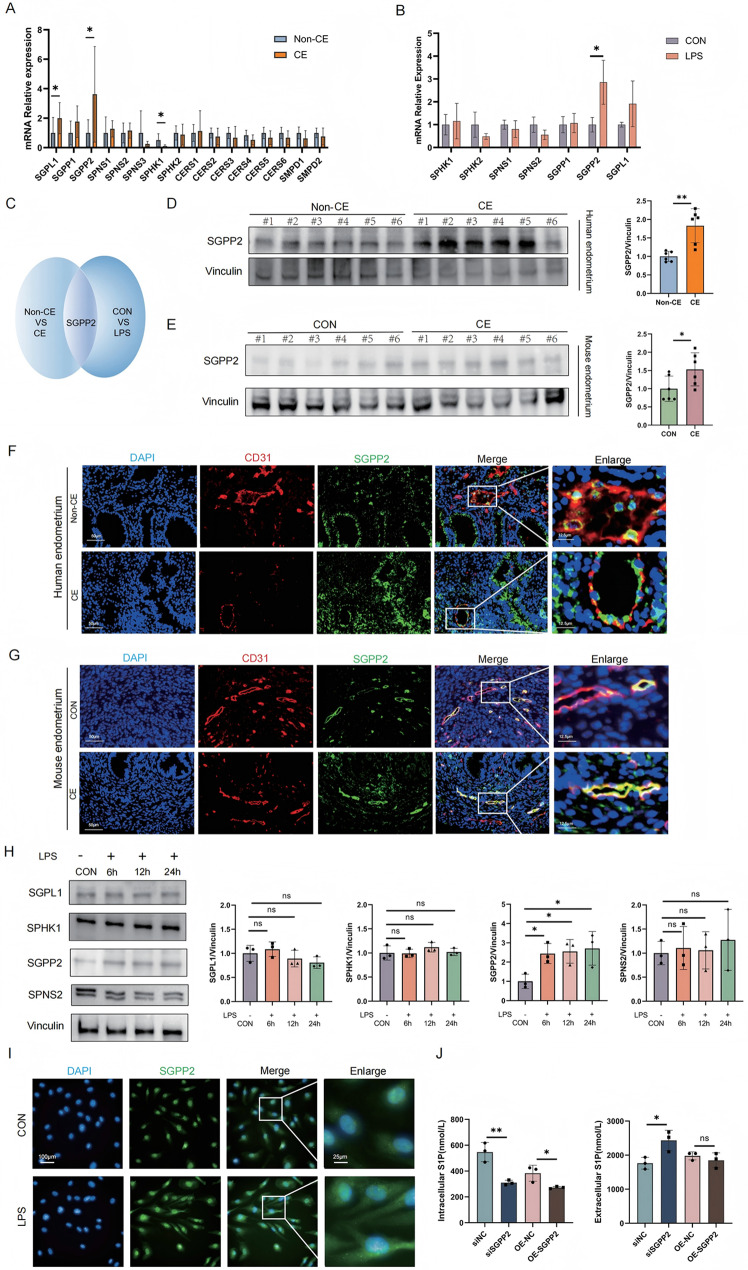
SGPP2 may be responsible for the decrease in S1P upon LPS stimulation. (**A**) Quantitative PCR (qPCR) analysis of S1P metabolic enzyme mRNA expression (SGPL1, SPHK1, SGPP1, SGPP2, SPNS2) in endometrial tissues from CE (*n* = 10) and non-CE (*n* = 10) patients, normalized to the reference gene B2M. (**B**) qPCR detected relative mRNA expression of S1P metabolic enzymes in HUVECs under LPS treatment compared to untreated control, normalized using B2M as a reference. (**C**) Venn diagram illustrating SGPP2 as the sole differentially expressed S1P metabolic enzyme identified in both human endometrial tissues (CE vs. non-CE) and HUVECs (LPS vs. CON). (**D**) Validation of SGPP2 changes in endometrial tissues from CE and non-CE patients via Western blot (*n* = 6), quantified using ImageJ software. (**E**) Validation of SGPP2 changes in mouse endometrial tissues from the control and CE model groups via Western blot (*n* = 6), quantified using ImageJ software. (**F**, **G**) Immunofluorescence staining of CD31 (red) and SGPP2 (green) in human and mouse endometrial tissues revealed that SGPP2 was primarily localized to vascular endothelial cells. (**H**) Western blot analysis of S1P metabolic enzymes (SGPL1, SPHK1, SGPP2, SPNS2) in HUVECs before and after LPS treatment, with band intensity quantification (ImageJ) to assess relative expression changes. (**I**) Immunofluorescence staining of SGPP2 (green**)** in HUVECs, visualizing endogenous SGPP2 expression and subcellular localization. (**J**) ELISA detection of changes in S1P and extracellular S1P levels changes upon treatment with siRNA targeting SGPP2 (siSGPP2) versus control (siNC), and overexpression plasmid targeting SGPP2 (OE-SGPP2) versus control (OE-NC) in HUVECs. All data are presented as mean ± SD. *P < 0.05, **P < 0.01; ns, no significant difference

To investigate the underlying cause of reduced S1P production in CE, we examined the expression of enzymes involved in sphingolipid metabolism. Our results showed that, compared with those in non-CE controls, the expression levels of sphingosine-1-phosphate lyase 1 (SGPL1), SPHK1, and SGPP2 were all significantly decreased in the endometrial tissues of CE patients (Fig. 4A). Furthermore, we assessed the expression of sphingolipid metabolic enzymes in vascular endothelial cells and found that SGPP2 was the only enzyme significantly upregulated in HUVECs following stimulation with LPS (Fig. 4B). Therefore, SGPP2 shows significant differences within tissues and cells (Fig. 4C). This observation was further confirmed by Western blot analysis (Fig. 4D, E, H). Additionally, immunofluorescence staining of human and mouse endometrial tissues for CD31(red, a marker of endothelial cells) and SGPP2 (green) revealed that SGPP2 was primarily localized to vascular endothelial cells (Fig. 4F, G). We also performed immunofluorescence localization of SGPP2 in HUVECs (Fig. 4I).

ELISA analysis of S1P levels in HUVECs following SGPP2 silencing by siRNA transfection demonstrated a significant decrease in intracellular S1P levels and increase in extracellular S1P levels (Fig. 4J). These results indicate that excess intracellular S1P is actively effluxed via transporters (such as SPNS2) to maintain S1P homeostasis in HUVECs. In contrast, SGPP2 overexpression markedly reduced S1P levels (Fig. 4J). Western blot confirmed the efficiency of SGPP2 knockout and overexpression (Figure S2A, B). These findings indicate that SGPP2 negatively regulates intracellular S1P homeostasis. Additionally, inflammatory responses of endometrium may upregulate SGPP2 expression in endothelial cells, thereby attenuating endogenous S1P production through enhanced degradation.

### CEBPD directly binds the SGPP2 promoter upon LPS stimulation in HUVECs

**Fig. 5:**
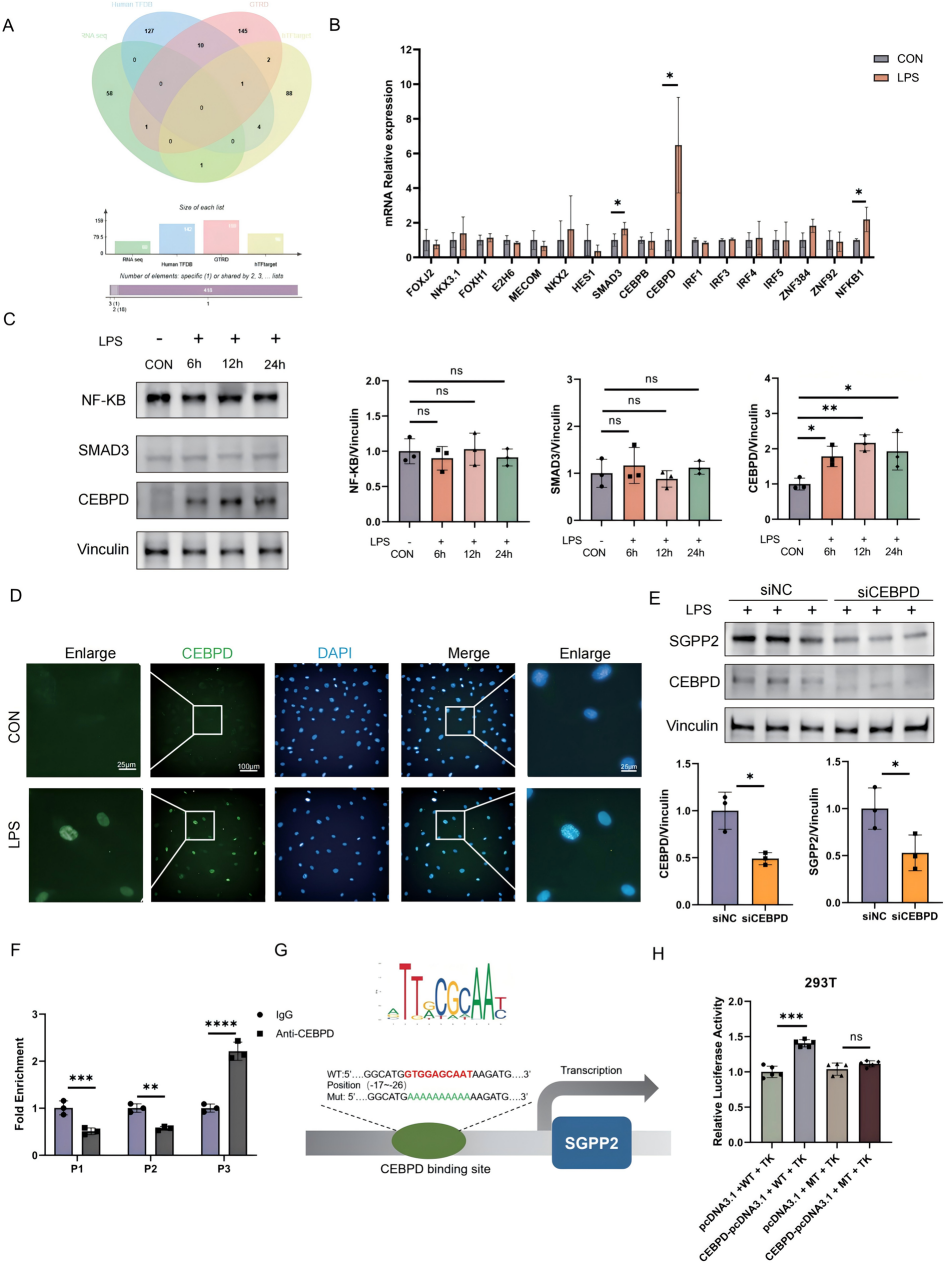
LPS-induced upregulation of *SGPP2* mediated by transcription factor CEBPD. (**A**) Integrated bioinformatics analysis to identify potential upstream transcription factors (TFs) regulating *SGPP2* expression. By combining RNA-seq data from LPS-treated HUVECs with the Human Transcription Factor Database (Human TFDB), Gene Transcription Regulation Database (GTRD), and hTFtarget database, candidate TFs were computationally predicted. (**B**) qPCR analysis of relative mRNA expression levels for 17 most probable candidate transcription factors in LPS-treated HUVECs, normalized to B2M as internal control. (**C**) Western blot was used to assess NF-κB, SMAD3, and CEBPD protein expression in HUVECs before and after LPS treatment. Band intensities were quantified by ImageJ software. (**D**) Immunofluorescence revealed significant nuclear translocation of CEBPD before and after LPS treatment (CEBPD: green, DAPI: blue). Images were captured at 20x magnification using high-content imaging. (**E**) Western blot validation of CEBPD and *SGPP2* protein expression in HUVECs treated with small interfering RNA targeting CEBPD (siCEBPD) or non-targeting control siRNA (siNC). Protein band intensities were quantified using ImageJ software. (**F**) In silico prediction of CEBPD binding sites within the *SGPP2* promoter using the JASPAR database, followed by chromatin immunoprecipitation (ChIP)-qPCR to experimentally validate direct binding of CEBPD to specific promoter regions. (**G**) Consensus binding motif of CEBPD (derived from JASPAR) and a schematic mechanism diagram illustrating the identified CEBPD binding sites within the *SGPP2* promoter region. (**H**) Dual-luciferase reporter assay assessing the transcriptional regulation of *SGPP2* by CEBPD. HUVECs were co-transfected with a CEBPD overexpression plasmid (CEBPD-pcDNA3.1) and reporter vectors containing either the wild-type (WT) *SGPP2* promoter or a site-mutant (MT) *SGPP2* promoter (with mutated CEBPD binding sites). Luciferase activity was measured to determine the functional impact of CEBPD on promoter activity. All data are presented as mean  ± SD. *P < 0.05, **P < 0.01, ***P < 0.001; ns, no significant difference

To identify nuclear transcription factors (TFs) that regulate SGPP2 expression in response to LPS stimulation, we integrated multi-omics data (including RNA-seq), the Human Transcription Factor Database (HTFD), the Gene Target Database, and annotated human transcriptional target information to systematically predict potential upstream TFs governing SGPP2 **(**Fig. 5A**)**. Based on predicted functional relevance, we prioritized the 17 most plausible candidate TFs and further evaluated their expression dynamics in HUVECs under LPS stimulation versus control group using RT-qPCR. Results demonstrated significant upregulation of SMAD3, CEBPD, and NF-κB1 in response to LPS stimulation **(**Fig. 5B**)**. However, Western blot analysis of protein expression revealed that among these three transcription factors, only CEBPD exhibited a marked increase following LPS treatment **(**Fig. 5C**)**. Immunofluorescence staining of HUVECs further confirmed that LPS stimulation induced a significant nuclear translocation of CEBPD **(**Fig. 5D**)**. Consistently, knockdown of CEBPD attenuated SGPP2 expression **(**Fig. 5E**)**. Functional validation by chromatin immunoprecipitation (ChIP) and dual-luciferase reporter assays subsequently demonstrated direct binding of CEBPD to the promoter region of SGPP2 **(**Fig. 5F-H**)**. We identifed CEBPD as a LPS-responsive transcription factor that directly regulates SGPP2 expression by binding to its promoter.

### LPS induces CEBPD-SGPP2 axis expression via the PERK-eIF2α pathway independent of ATF4

**Fig. 6:**
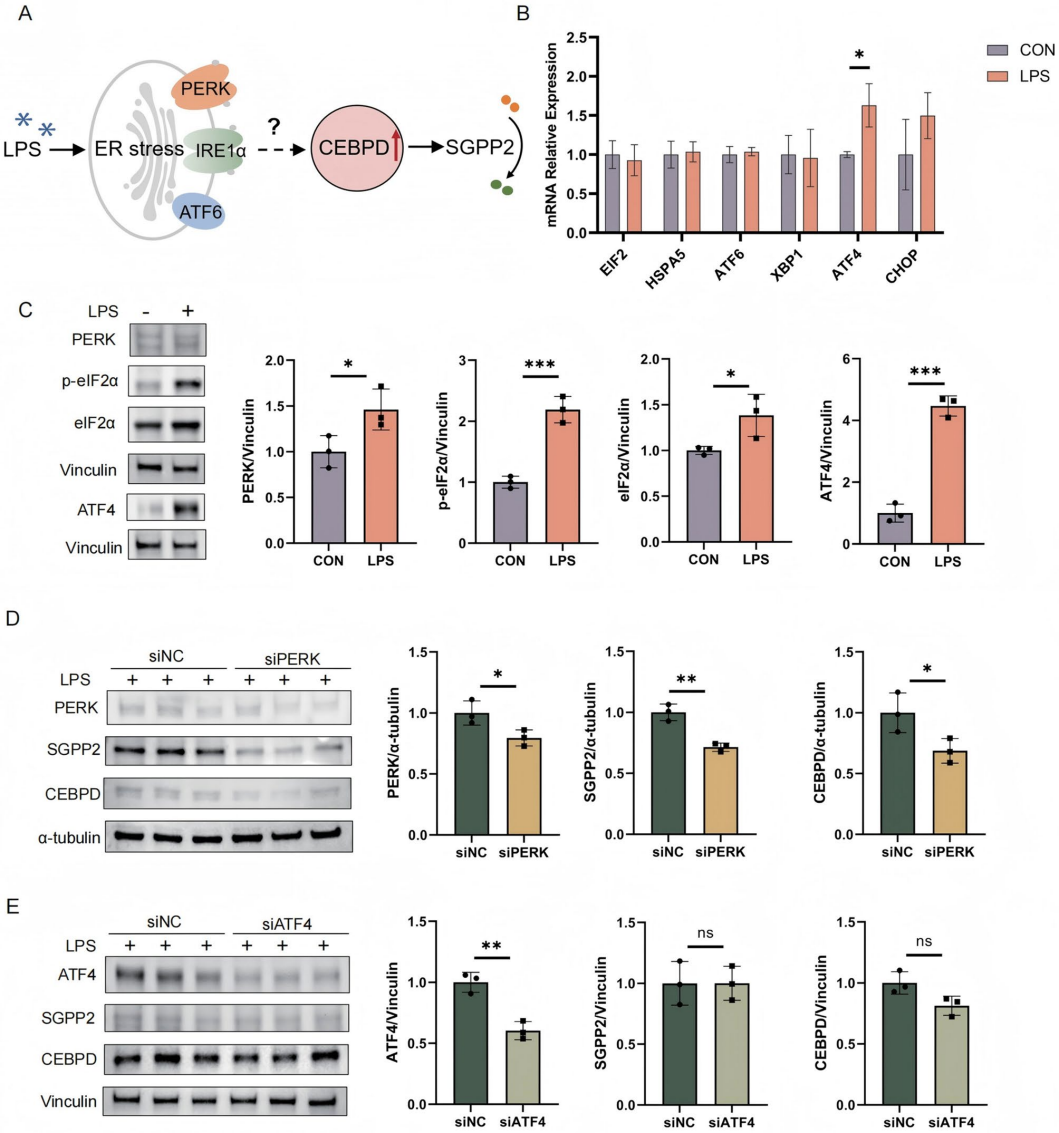
LPS-induced endoplasmic reticulum stress mediates CEBPD-SGPP2 upregulation via the PERK pathway. (**A**) Schematic model hypothesizing that LPS stimulation may induce ER stress in HUVECs, leading to upregulation of the CEBPD-SGPP2 axis. (**B**) qPCR analysis of ER stress-related molecular markers in HUVECs under LPS treatment (1 µg/mL, 24 h). Relative mRNA expression levels of key ER stress indicators were normalized to the reference gene B2M. (**C**) Western blot validation of PERK-eIF2α-ATF4 signaling pathway activation in HUVECs before and after LPS treatment. Protein expression levels of pathway components (PERK, eIF2α, p-eIF2α, ATF4) were quantified using ImageJ software to confirm the engagement of the canonical ER stress sensor pathway. (**D**) Western blot analysis of CEBPD and SGPP2 protein levels in HUVECs upon LPS stimulation, comparing cells transfected with siRNA targeting PERK (siPERK) versus siNC. Protein band intensities were quantified using ImageJ software. (**E**) Under LPS stimulation, changes in CEBPD and SGPP2 were verified by Western blot after treatment with ATF4-targeting siRNA (siATF4) compared to siNC. Protein band intensities were quantified using ImageJ software. All data are presented as mean ± SD. *P < 0.05, **P < 0.01, ***P < 0.001; ns, no significant difference

It has been demonstrated that the expression of CEBPD can be activated under conditions of ER stress in tumor cells [34]. Based on these findings, we hypothesized that LPS stimulation induces ER stress in HUVECs, which in turn leads to the upregulation of the CEBPD-SGPP2 axis **(**Fig. 6A**)**.

To test this hypothesis, we first performed qPCR to examine the expression of the UPR markers (eIF2α, HSPA5, ATF6, XBP1, ATF4, CHOP) involved in the three canonical branches. Among these, we observed a significant upregulation of ATF4 (Fig. 6B), a downstream effector of the PERK pathway. Accordingly, as shown in Fig. 6C, PERK, phosphorylation of eIF2α and ATF4 expression were strongly increased in response to LPS (Fig. 6C). These data demonstrate LPS activates the PERK-eIF2α-ATF4 signaling pathway.

To further investigate the role of the PERK pathway in mediating the expression of CEBPD and SGPP2 under inflammation, we performed siRNA-mediated knockdown of PERK in HUVECs. The results showed that, compared with the control group, CEBPD expression and SGPP2 expression were significantly decreased in siPERK group (Fig. 6D). In contrast, in the siATF4 group, knockdown of ATF4 did not significantly alter the LPS-induced expression of either CEBPD or SGPP2 (Fig. 6E). In conclusion, these findings indicate that LPS triggers ER stress and modulates the expression of the CEBPD-SGPP2 axis mainly through the PERK-eIF2α signaling pathway, independently of ATF4..

### S1P attenuates LPS-induced endometrial inflammation and vascular dysfunction to improve pregnancy outcomes in a mouse model of endometritis

**Fig. 7:**
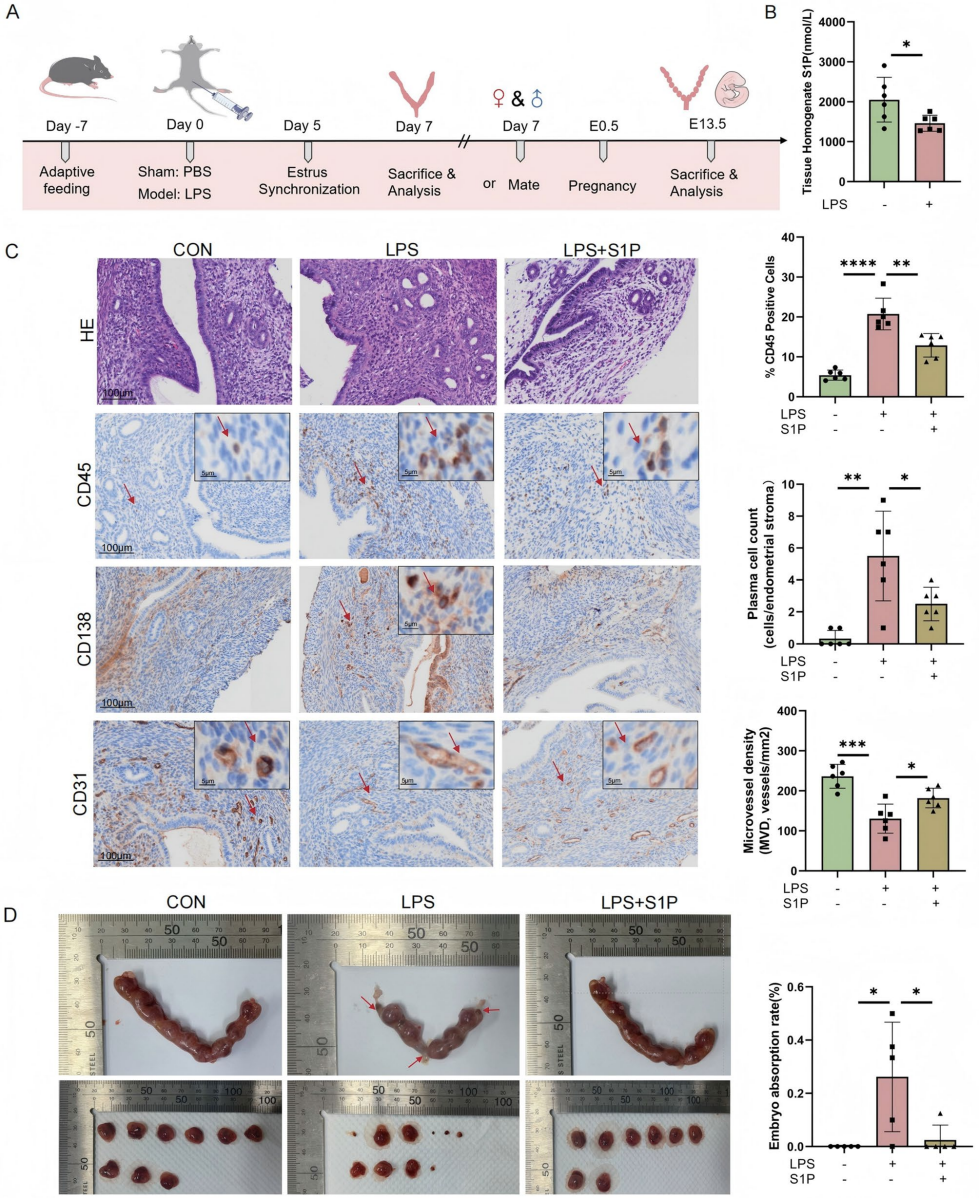
S1P rescues LPS-induced endometrial inflammation and vascular dysfunction to improve pregnancy outcomes in an endometritis mouse model. (**A**) The endometritis mouse model was established via intrauterine LPS injection. Mice treated with LPS + S1P served as the rescue group. Female mice were mated with males, and endometrial tissue was collected on day 7 post-treatment. The day of implantation was recorded as E0.5, and pregnancy outcomes were observed and recorded at E13.5. (**B**) S1P levels in endometrial tissue from endometritis mouse models were quantified **by** ELISA (*n* = 6). (**C**) HE staining and CD45/CD138/CD31 expression via immunohistochemistry in control, LPS, and LPS + S1P groups were observed. Quantitative analysis of positive cell percentage and cell count was performed using the HALO Image Analysis System (*n* = 6). (**D**) Typical macroscopic morphological comparisons of uterine and embryonic conditions in control, LPS, and LPS + S1P groups are shown, and statistical analysis of embryo absorption rates (*n* = 5) is presented. All data are presented as mean ± SD. *P < 0.05, **P < 0.01, ***P < 0.001; ns, no significant difference. CON: control

To further evaluate the impact of S1P on pregnancy outcomes, we established a mouse model of endometritis via intrauterine LPS injection (Fig. 7A). ELISA assays revealed a significant reduction in endometrial S1P levels in the LPS-induced inflammatory model, consistent with observations in human endometrial tissues and HUVECs (Fig. 7B). Seven days after LPS stimulation, CD45⁺ monocytes were observed to accumulate in the endometrial stroma. Furthermore, the number of CD138⁺ plasma cells in the endometrial stromal was significantly increased in the LPS-treated group, whereas S1P co-administration (LPS + S1P group) significantly reduced these immune cells infiltration, suggesting that S1P alleviates LPS-induced endometrial inflammation (Fig. 7C).

Furthermore, IHC staining of CD31 showed significant decrease of MVD after LPS treatment. However, this defect was rescued by S1P treatment (Fig. 7C). Following LPS treatment, the rate of embryo absorption was significantly increased compared to the control group. In contrast, the embryo resorption rate was significantly lower in the LPS + S1P group than in the LPS group (Fig. 7D). In summary, S1P supplementation effectively counteracted inflammation-induced angiogenesis impairment and pregnancy loss.

## Discussion

Here, we reveal a critical signaling pathway whereby CE induces vascular dysfunction through ER stress. Chronic inflammation in the endometrium activates ER stress, which subsequently triggers the activation of CEBPD via the PERK-eIF2α pathway. Activated CEBPD upregulates the expression of SGPP2, which disrupts the metabolic homeostasis of S1P, leading to reduced S1P levels. The decrease in S1P availability inhibits its capacity to activate the STAT3-VEGFA axis through S1PR1, thereby impairing endometrial angiogenesis and compromising pregnancy outcome. This mechanistic cascade provides a novel molecular perspective for understanding the pathological basis of adverse reproductive outcomes in patients with CE. These findings highlight the critical role of the CEBPD-SGPP2-S1P axis in mediating adverse reproductive outcomes in CE.

Blood vessels are important players in the inflammatory process. Consistent with our observations, alterations in blood vessels in the endometrium of CE patients were studied by Carvalho. In 85.7% of cases, vascular changes were associated with CE, while CE without vascular changes was observed in only 7.3% of cases [10]. Furthermore, we identified a specific reduction in S1P levels in both CE patient tissues and an inflammation model of LPS-induced HUVECs. Our study strongly suggests that S1P metabolic dysfunction serves as a pivotal link connecting inflammation and vascular dysfunction. S1P is well-established to maintain vascular homeostasis by promoting endothelial cell survival, migration, and lumen formation [35, 36]. We further explored the functional mechanisms of S1P within endothelial cells. Experimental results unequivocally demonstrated that exogenous S1P supplementation effectively reversed LPS-induced impairments in tube formation, migration, and invasion capacities in HUVECs. In conclusion, these findings indicate that the reduction in S1P levels observed in CE patients represents a critical factor contributing to impaired angiogenesis.

S1P regulates endothelial function by binding to specific S1PR. We identified S1PR1 as the most abundantly expressed S1P receptor in HUVECs. Previous studies have demonstrated that S1PR1 knockout leads to incomplete vascular maturation, characterized by embryonic hemorrhage and subsequent intrauterine lethality in mice [37, 38]. This demonstrates the critical importance of S1PR1 for normal vascular function during pregnancy. However, previous studies have yielded seemingly paradoxical findings regarding the role of S1PR1 in regulating VEGFA expression: while certain investigations demonstrated that S1PR1 inhibits VEGFA expression [39, 40], others identified a promotional effect of S1PR1 on VEGFA expression [41, 42]. These contradictory observations suggest that the S1P/S1PR1 axis exhibits context-dependent regulatory effects on angiogenesis, functioning as either a pro-angiogenic or anti-angiogenic mediator depending on the specific disease microenvironment.

Under physiological conditions, the level of S1P is in a dynamic equilibrium and is regulated by various sphingolipid metabolic enzymes. Sphingolipid metabolic enzymes exhibit cell-type-specific variation. We found that SGPP2 is mainly expressed in blood vessels. Intracellular S1P levels are dynamically regulated by its synthetic enzymes (such as sphingosine kinases SPHK1/2) and degradative enzymes (including S1P lyase and S1P phosphatases). Among these, S1P phosphatases (such as SGPP1 and SGPP2) mediate the dephosphorylation of S1P to generate sphingosine [17, 18]. Through multiple validations using clinical samples and cellular models, our research found that under inflammatory conditions, the expression of SGPP2 in endometrial blood vessels significantly increased. Several reports show that the LPS-induced increase in SGPP2 expression is consistent with our result [43, 44]. These findings indicate that normal endothelial cell function critically depends on the precise regulation of S1P concentration. Any disruption to the balance of sphingolipid metabolism may lead to vascular dysfunction.

SGPP2 is a key enzyme in the sphingolipid metabolism pathway and plays an important regulatory role in physiological processes such as cell proliferation, immune regulation. Recent studies have found that SGPP2 is abnormally expressed in inflammatory diseases and various tumors. Its expression level is closely related to disease progression, suggesting that SGPP2 has dual value as a biomarker and a potential therapeutic target. In inflammation-related diseases, the expression of SGPP2 is significantly upregulated [44]. For example, in the inflamed colonic mucosa of patients with ulcerative colitis, the expression level of SGPP2 was significantly higher than that of normal tissue. Functional experiments further showed that SGPP2 deficiency could inhibit apoptosis of intestinal epithelial cells and enhance the integrity of the mucosal barrier [45], indicating that SGPP2 has a direct regulatory role in the inflammatory response. In terms of tumors, Studies have shown that SGPP2 can promote the development of tumors such as lung adenocarcinoma and gastric cancer, and its expression level is positively correlated with clinicopathological features such as lymph node metastasis, suggesting that SGPP2 may be an important indicator for assessing tumor prognosis [46–48].

The regulatory mechanisms governing SGPP2 expression appear to be stimulus-dependent. While previous studies indicated that SGPP2 expression is regulated by NF-κB upon TNF-α stimulation, our findings in LPS-stimulated HUVECs reveal that SGPP2 transcription is mediated by CEBPD rather than NF-κB. This discrepancy highlights the context-dependent nature of SGPP2 gene regulation. CEBPD is a member of a transcription factor family, which also includes C/EBPα, β, -γ, and -ϵ, as well as C/EBP homologous protein (CHOP). Inflammation is an important factor in ER stress [49, 50]. CEBPD is induced under stress stimuli (such as cytokine stimulation, LPS, corticosteroids, radiation, and hypoxia) [51–53]. Recently, there has been a report showing that the expression of CEBPD is induced by ER stress [34]. The ER is the primary site for protein synthesis, folding, and post-translational processing, as well as the location for de novo sphingolipid synthesis and the sphingolipid cycle pathway [18]. This indicates that alterations in the CEBPD-SGPP2 signaling axis under LPS stimulation may be associated with ER stress. Our research found that LPS stimulation of HUVECs can induce the PERK-eIF2α-ATF4 pathway, inducing ER stress. Consistently, we further observed that knockout of PERK led to significant downregulation of both CEBPD and SGPP2 expression, whereas ATF4 knockout had no detectable effect on their expression levels. Similar to the findings of Namratha Sheshadri et al., CEBPD upregulation in ER stress does not depend on ATF4 [34]. Together, these results suggest that CEBPD expression is independent of typical ATF4-dependent ER stress signaling.

To investigate the pathophysiological relevance of the S1P pathway, We established a murine endometritis model by injecting LPS into the uterus, characterized by CD45^+^ immune cells and CD138^+^ plasma cells infiltration. The addition of S1P as a drug not only improved the defects of endometrial blood vessels but also decreased the embryo resorption rate. These finding provided strong preclinical evidence for targeted therapeutic intervention against reproductive dysfunction related to CE using S1P. In summary, as a critical enzyme regulating S1P homeostasis, SGPP2 plays a crucial role in the progression of diseases such as inflammation and tumors. It not only serves as a potential biomarker for assessing disease progression but also provides a novel molecular target for developing corresponding therapeutic strategies. Further research is needed to explore the regulatory mechanisms and pathological functions of SGPP2 to promote its clinical application.

However, this study still has the following limitations. Although the LPS model simulates the inflammatory characteristics of endometritis, it differs from the pathological conditions of human endometritis. Additionally, this study is primarily based on mouse models and HUVECs. In the future, it is necessary to verify these findings in human primary endometrial endothelial cells. Moreover, the metabolism of S1P involves multiple enzymes and receptors, which forms a highly complex regulatory network. The interaction between SGPP2 and other enzymes (such as SPHK1, SGPL1) also deserves further study.

## Conclusion

**Fig. 8:**
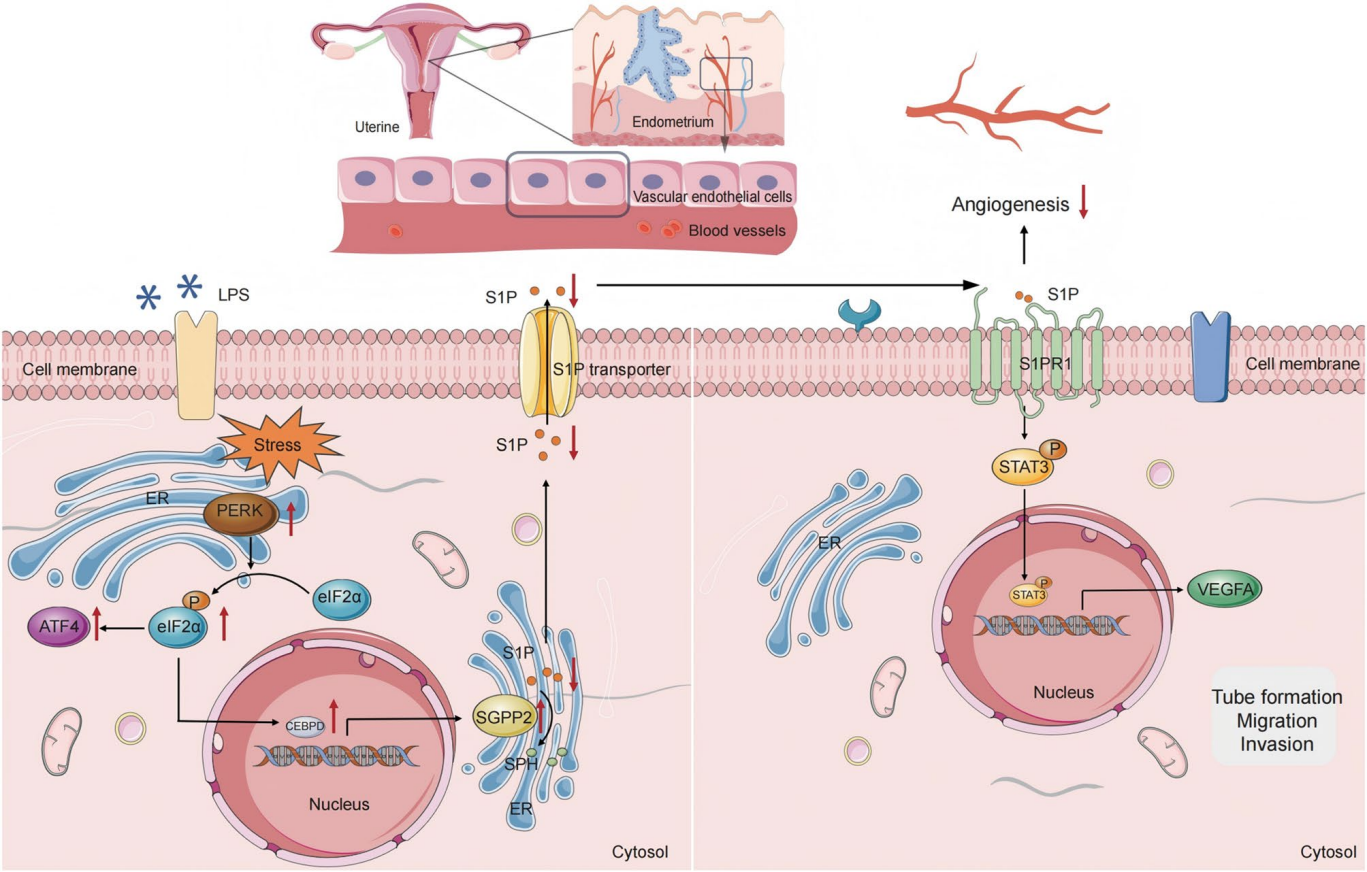
Schematic diagram of inflammation-induced vascular dysfunction by ER stress-driven sphingolipid reprogramming

In summary, our findings define a signaling cascade where inflammation triggers vascular dysfunction via ER stress-driven sphingolipid reprogramming (Fig. 8). Inflammation in endometrium activates CEBPD, which mediates PERK/ER stress-induced SGPP2 expression in endothelial Cells, leading to angiogenesis dysfunction and reproductive failure. These findings fill a critical gap in the molecular understanding of vascular dysfunction in CE. Modulating S1P metabolism or blocking upstream ER stress signals may restore endometrial vascular integrity and improve pregnancy outcomes.

## Supplementary information

Below is the link to the electronic supplementary material.

Supplementary Material 1

Supplementary Material 2

Supplementary Material 3

## Data Availability

The original contributions presented in the study are included in the article supplementary material. Further inquiries are available from the corresponding authors upon reasonable request.
