# Supplementary material for: CEBPD-mediated SGPP2 upregulation via PERK/ER stress in endothelial cells disrupts S1P homeostasis and impairs angiogenesis in chronic endometritis
Source: J Transl Med. 2025 Dec 17;24:98. doi: 10.1186/s12967-025-07558-0 (PMC12822190; doi:10.1186/s12967-025-07558-0)
Supplement: Supplementary file 3 — Supplementary Material 3 [file 12967_2025_7558_MOESM3_ESM.pdf]

Figure 2G

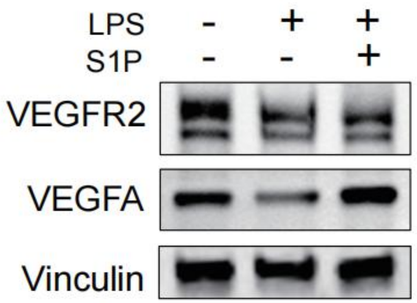
LPS - + +
S1P - - +
VEGFR2
VEGFA
Vinculin

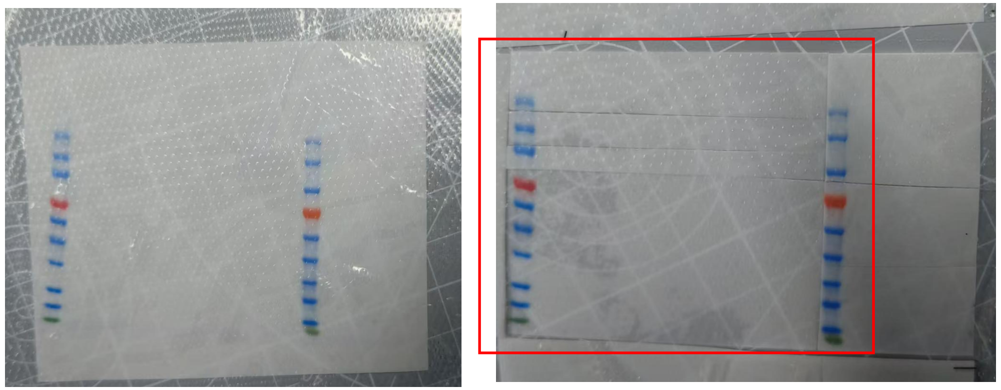

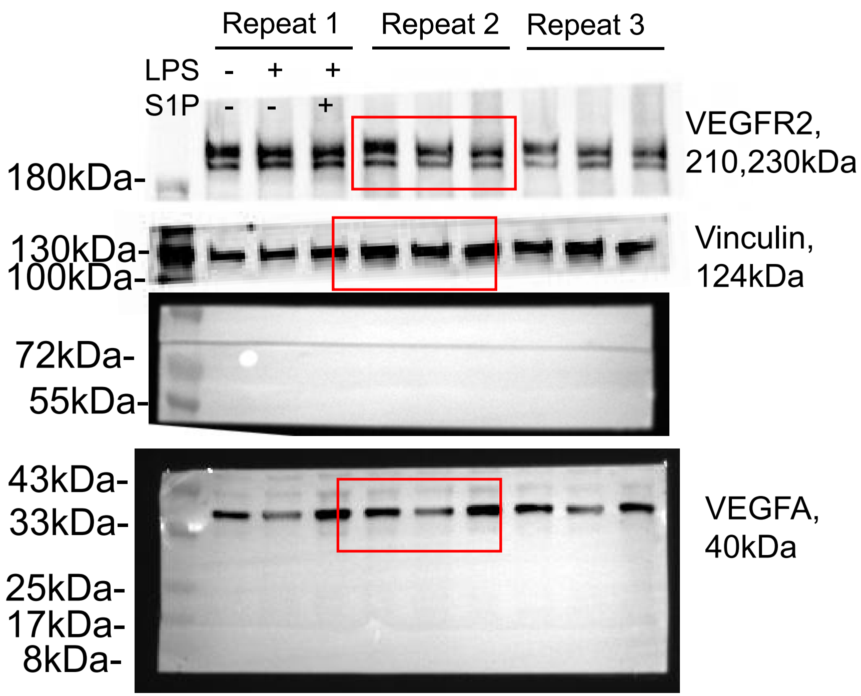
Repeat 1
Repeat 2
Repeat 3
LPS - + +
S1P - - +
VEGFR2,
210,230kDa
180kDa-
Vinculin,
124kDa
130kDa-
100kDa-
72kDa-
55kDa-
43kDa-
33kDa-
VEGFA,
40kDa
25kDa-
17kDa-
8kDa-

# Figure 3G

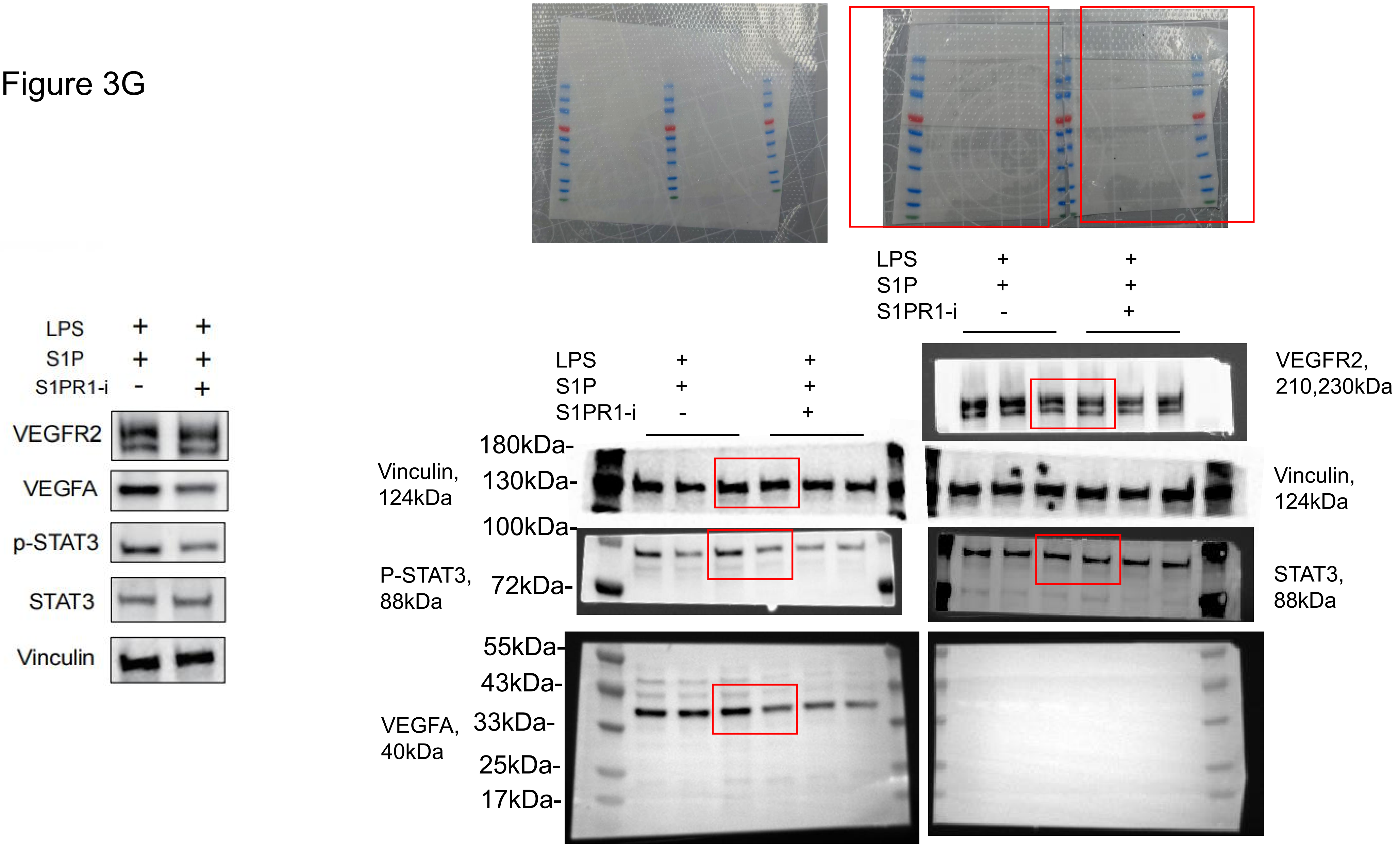

LPS + +
S1P + +
S1PR1-i - +
VEGFR2
VEGFA
p-STAT3
STAT3
Vinculin
LPS + +
S1P + +
S1PR1-i - +
VEGFR2,
210,230kDa
Vinculin,
124kDa
STAT3,
88kDa
LPS + +
S1P + +
S1PR1-i - +
180kDa-
130kDa-
100kDa-
72kDa-
Vinculin,
124kDa
P-STAT3,
88kDa
55kDa-
43kDa-
33kDa-
25kDa-
17kDa-
VEGFA,
40kDa

# Figure 3H

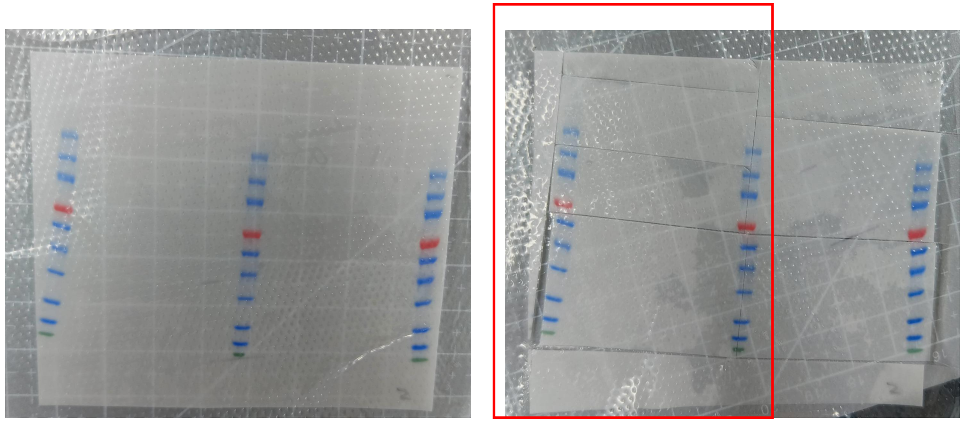

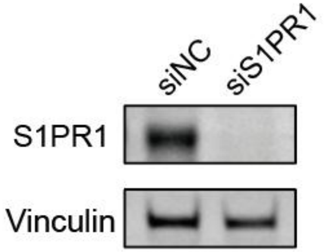
siNC
siS1PR1
S1PR1
Vinculin

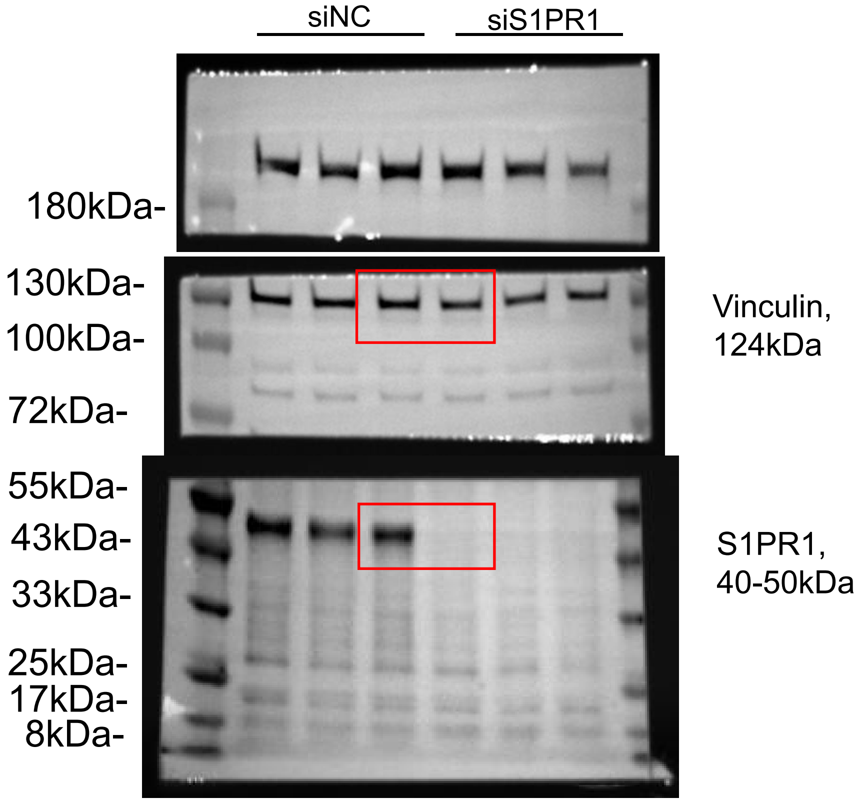
siNC
siS1PR1
180kDa-
130kDa-
100kDa-
72kDa-
55kDa-
43kDa-
33kDa-
25kDa-
17kDa-
8kDa-
Vinculin,
124kDa
S1PR1,
40-50kDa

Figure 3I

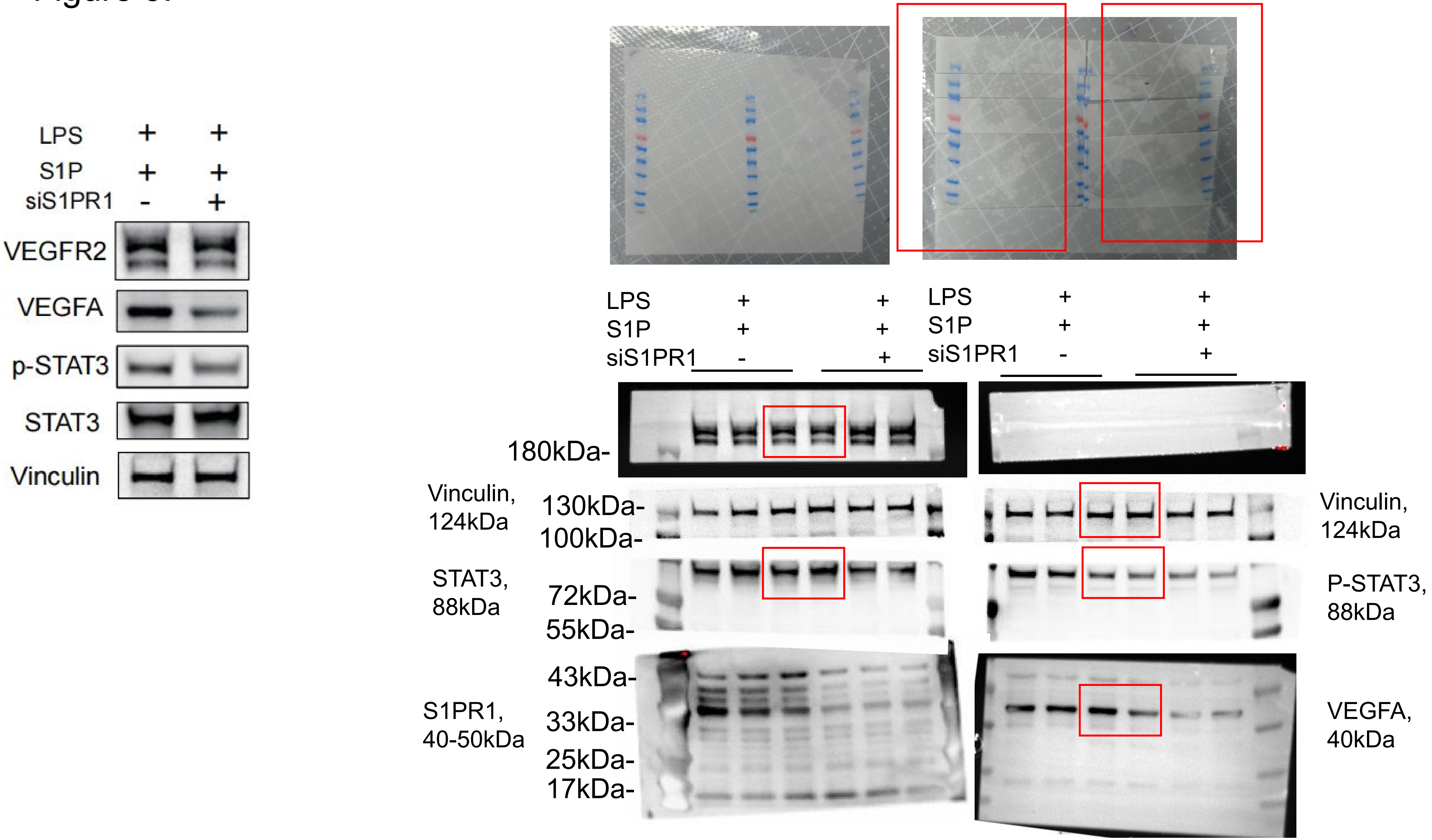
LPS + +
S1P + +
siS1PR1 - +
VEGFR2
VEGFA
p-STAT3
STAT3
Vinculin
LPS + +
S1P + +
siS1PR1 - +
LPS + +
S1P + +
siS1PR1 - +
180kDa-
Vinculin, 124kDa
130kDa-
100kDa-
STAT3, 88kDa
72kDa-
55kDa-
43kDa-
S1PR1, 40-50kDa
33kDa-
25kDa-
17kDa-
Vinculin, 124kDa
P-STAT3, 88kDa
VEGFA, 40kDa

Figure 4D

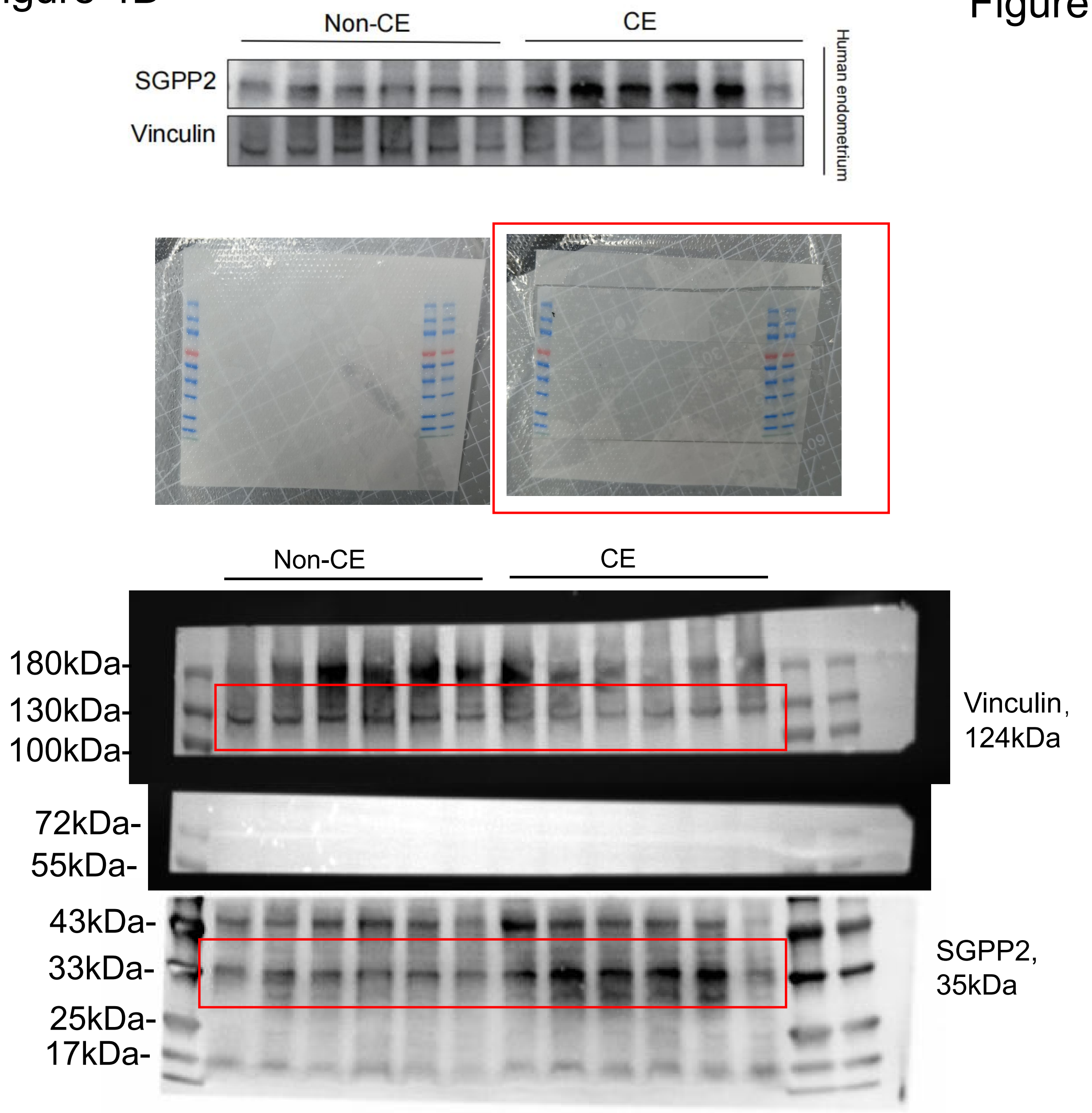


Figure 4E

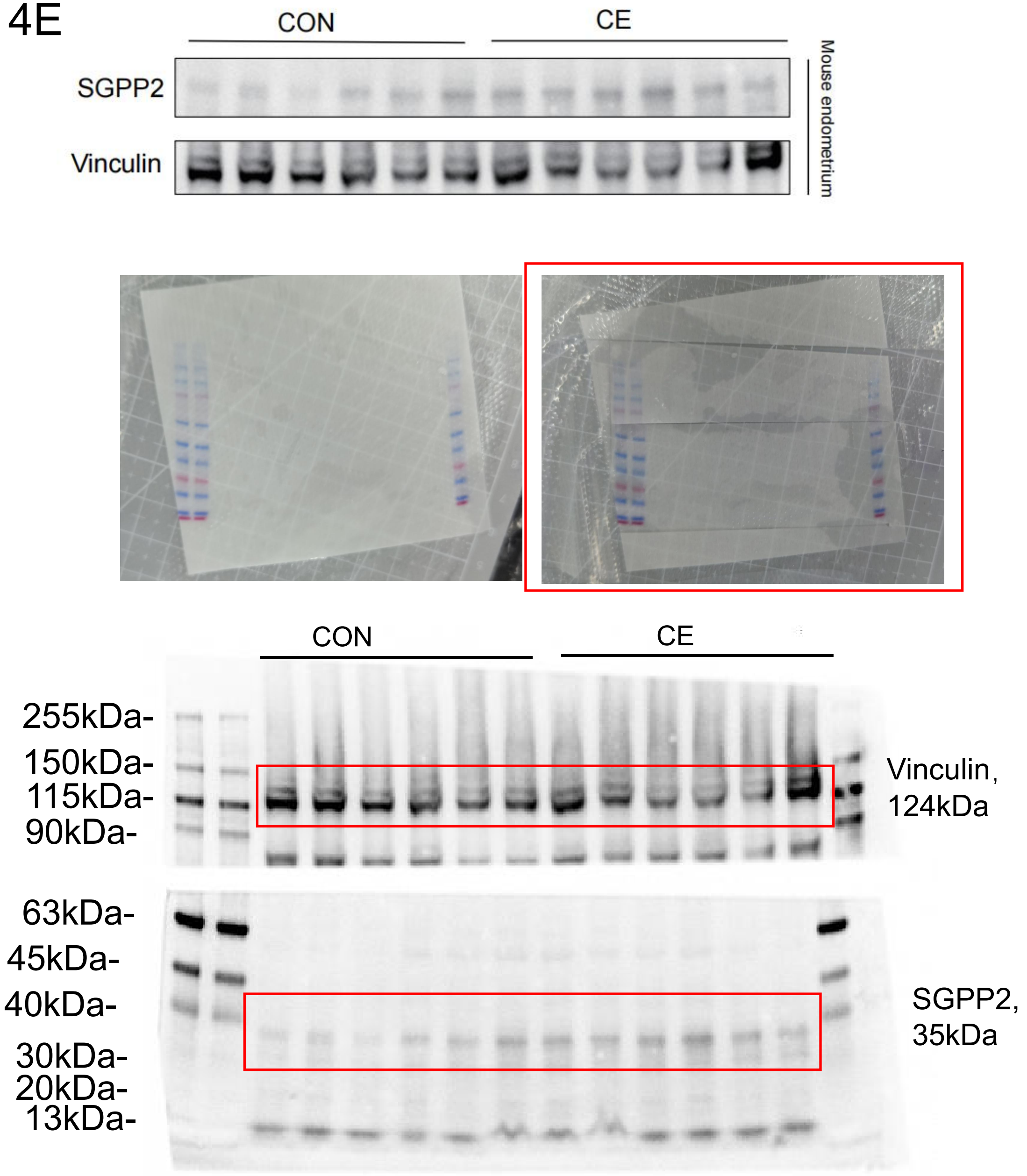

# Figure 4H

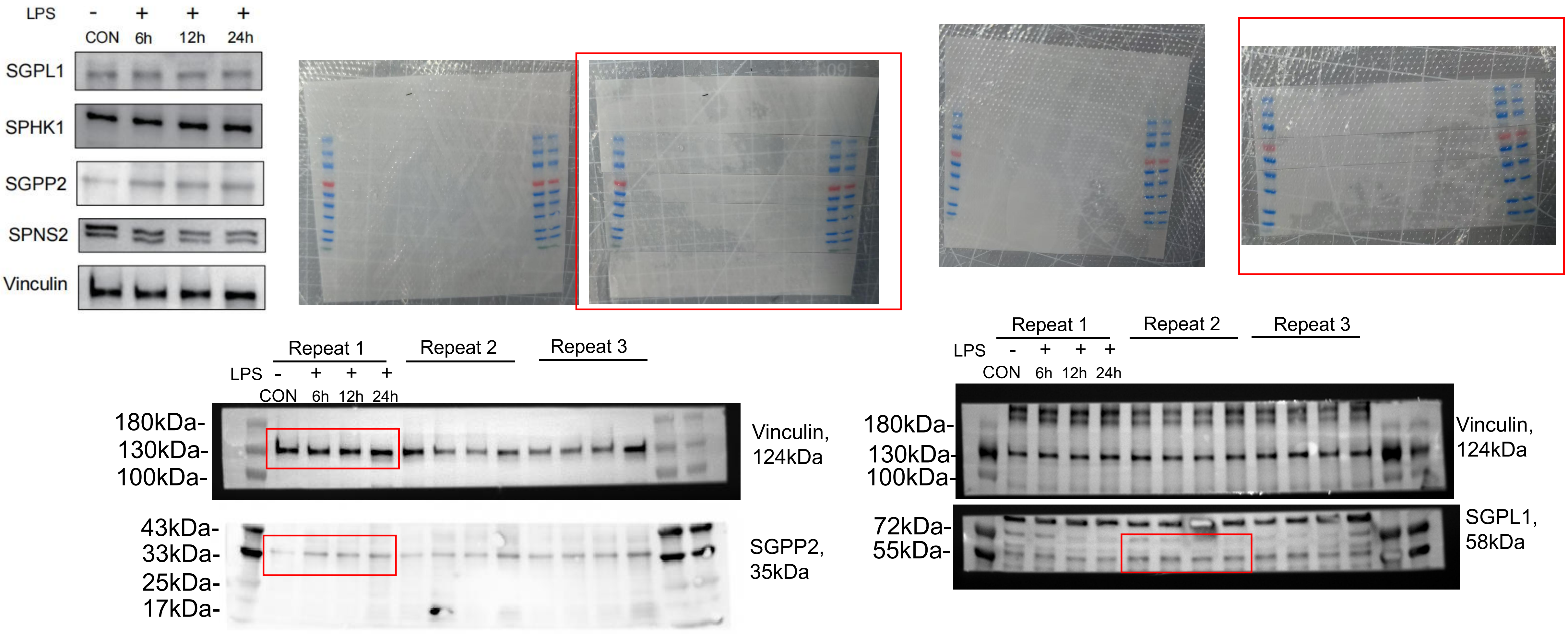

LPS
-
+
+
+
CON
6h
12h
24h
SGPL1
SPHK1
SGPP2
SPNS2
Vinculin
Repeat 1
Repeat 2
Repeat 3
LPS
-
+
+
+
CON
6h
12h
24h
180kDa-
130kDa-
100kDa-
Vinculin,
124kDa
43kDa-
33kDa-
25kDa-
17kDa-
SGPP2,
35kDa
Repeat 1
Repeat 2
Repeat 3
LPS
-
+
+
+
CON
6h
12h
24h
180kDa-
130kDa-
100kDa-
Vinculin,
124kDa
72kDa-
55kDa-
SGPL1,
58kDa

# Figure 4H

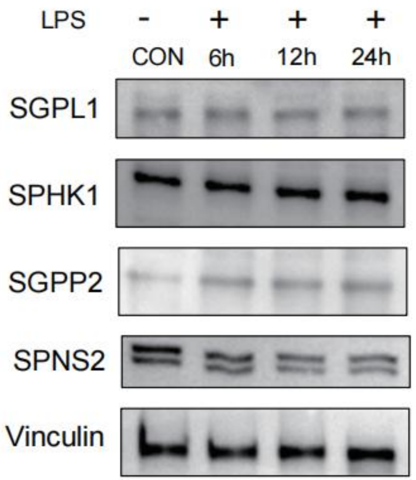

LPS - + + +
CON 6h 12h 24h
SGPL1
SPHK1
SGPP2
SPNS2
Vinculin


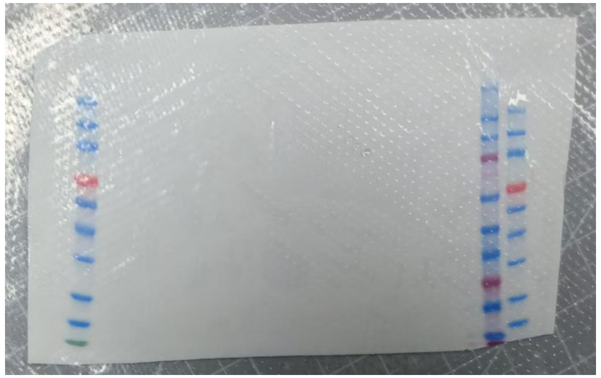

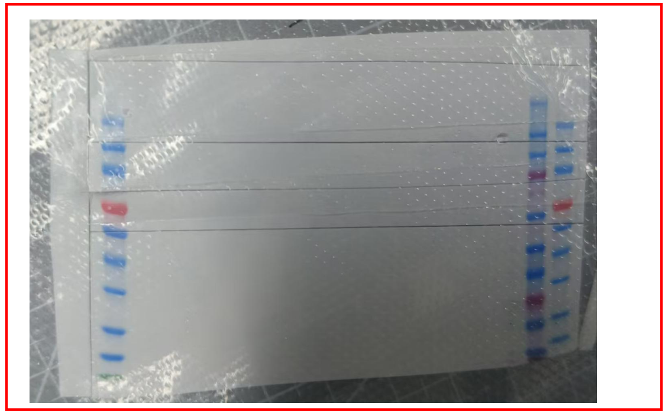

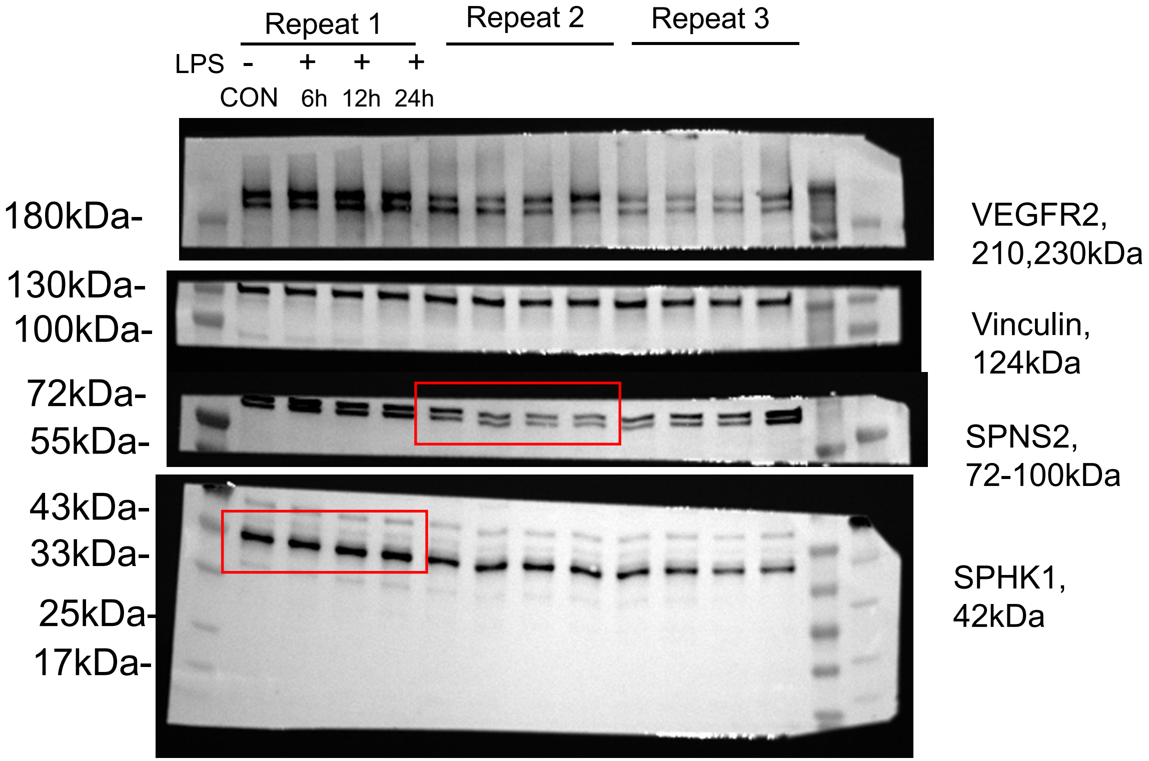

Repeat 1
Repeat 2
Repeat 3
LPS - + + +
CON 6h 12h 24h
180kDa-
130kDa-
100kDa-
72kDa-
55kDa-
43kDa-
33kDa-
25kDa-
17kDa-
VEGFR2, 210,230kDa
Vinculin, 124kDa
SPNS2, 72-100kDa
SPHK1, 42kDa

# Figure 5C

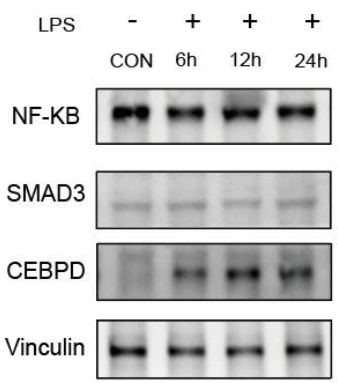

LPS - + + +
CON 6h 12h 24h
NF-KB
SMAD3
CEBPD
Vinculin


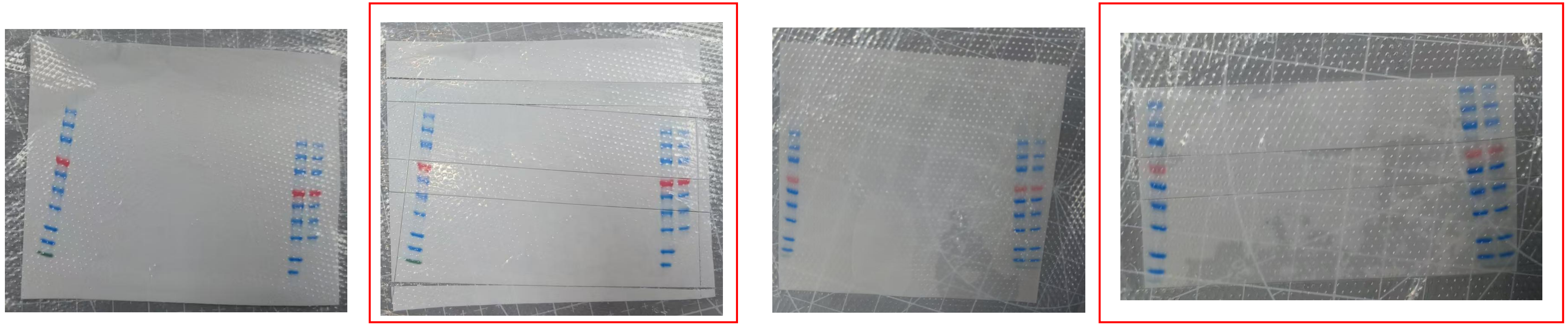

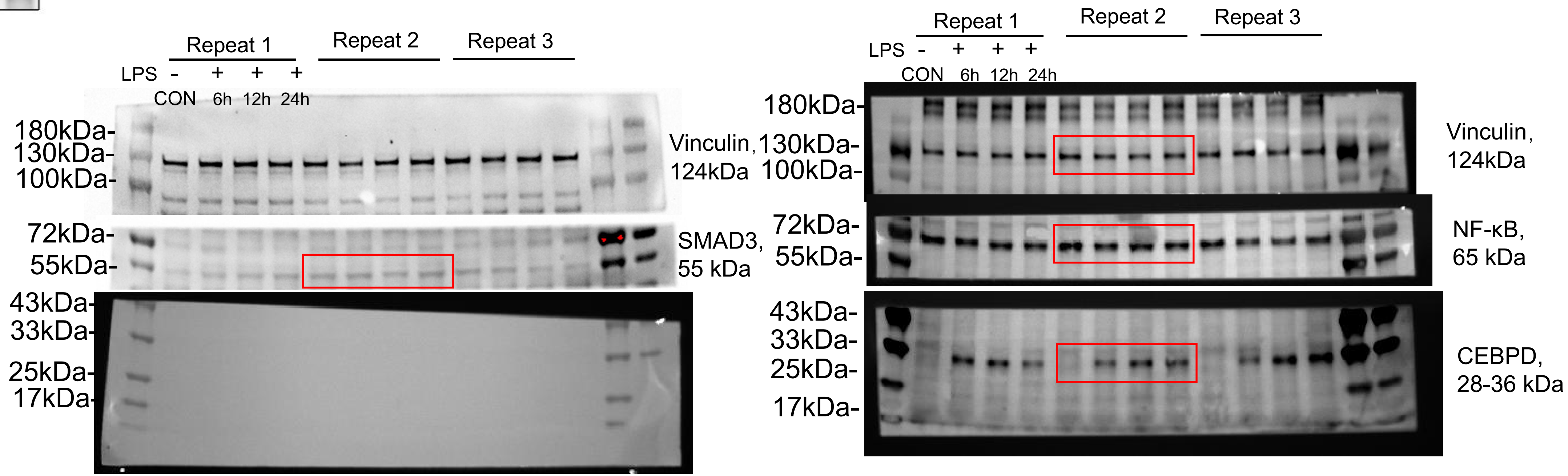

Repeat 1
Repeat 2
Repeat 3
LPS - + + +
CON 6h 12h 24h
180kDa-
130kDa-
100kDa-
Vinculin, 124kDa
72kDa-
55kDa-
SMAD3, 55 kDa
43kDa-
33kDa-
25kDa-
17kDa-
Repeat 1
Repeat 2
Repeat 3
LPS - + + +
CON 6h 12h 24h
180kDa-
130kDa-
100kDa-
Vinculin, 124kDa
72kDa-
55kDa-
NF-κB, 65 kDa
43kDa-
33kDa-
25kDa-
17kDa-
CEBPD, 28-36 kDa

Figure 5E

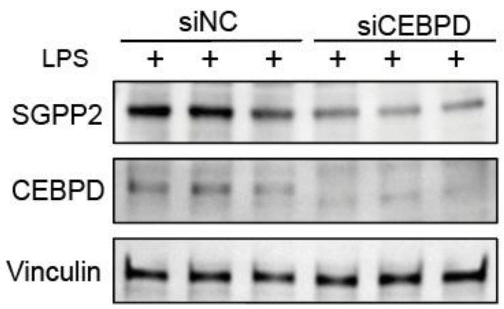

siNC
siCEBPD
LPS
+ + + + + +
SGPP2
CEBPD
Vinculin


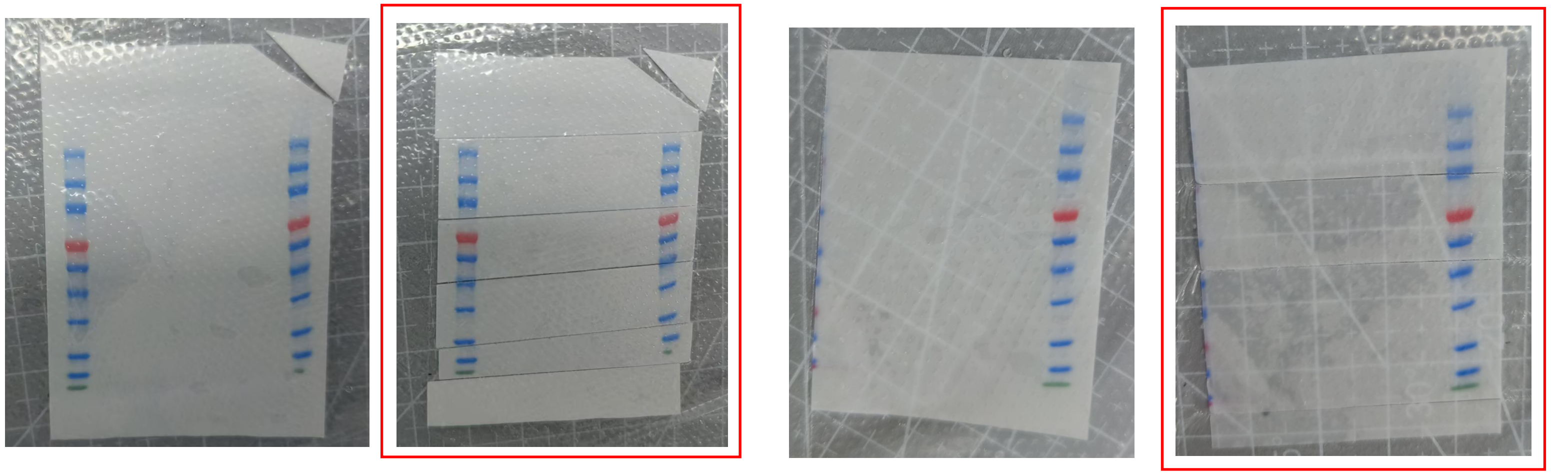

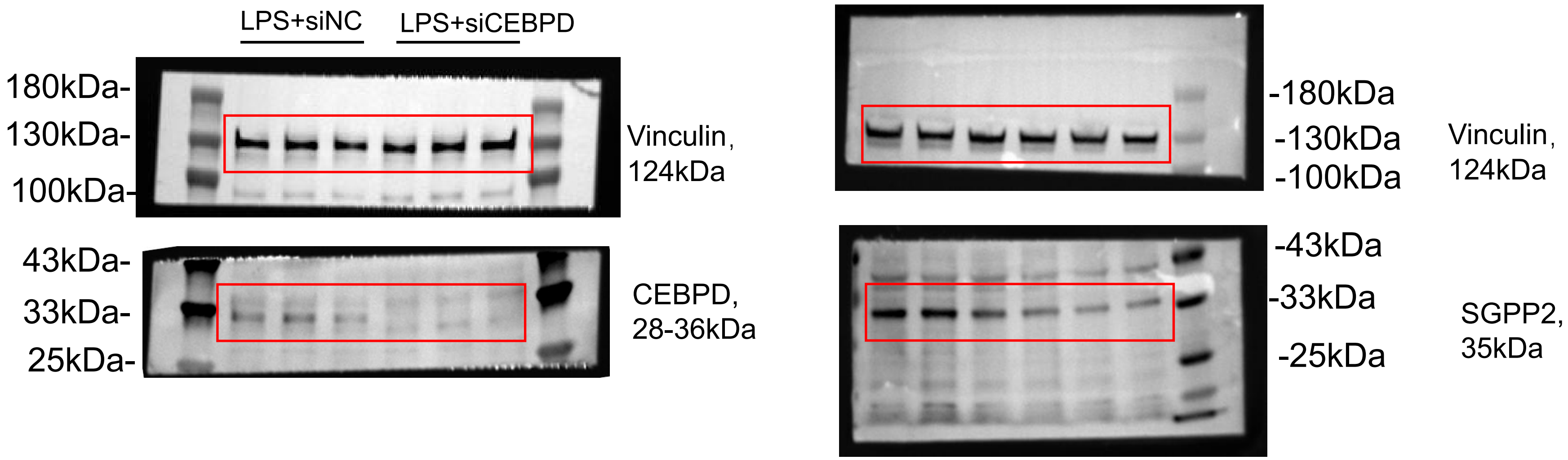

LPS+siNC
LPS+siCEBPD
180kDa-
130kDa-
100kDa-
Vinculin, 124kDa
43kDa-
33kDa-
25kDa-
CEBPD, 28-36kDa
-180kDa
-130kDa
-100kDa
Vinculin, 124kDa
-43kDa
-33kDa
-25kDa
SGPP2, 35kDa

Figure 6C

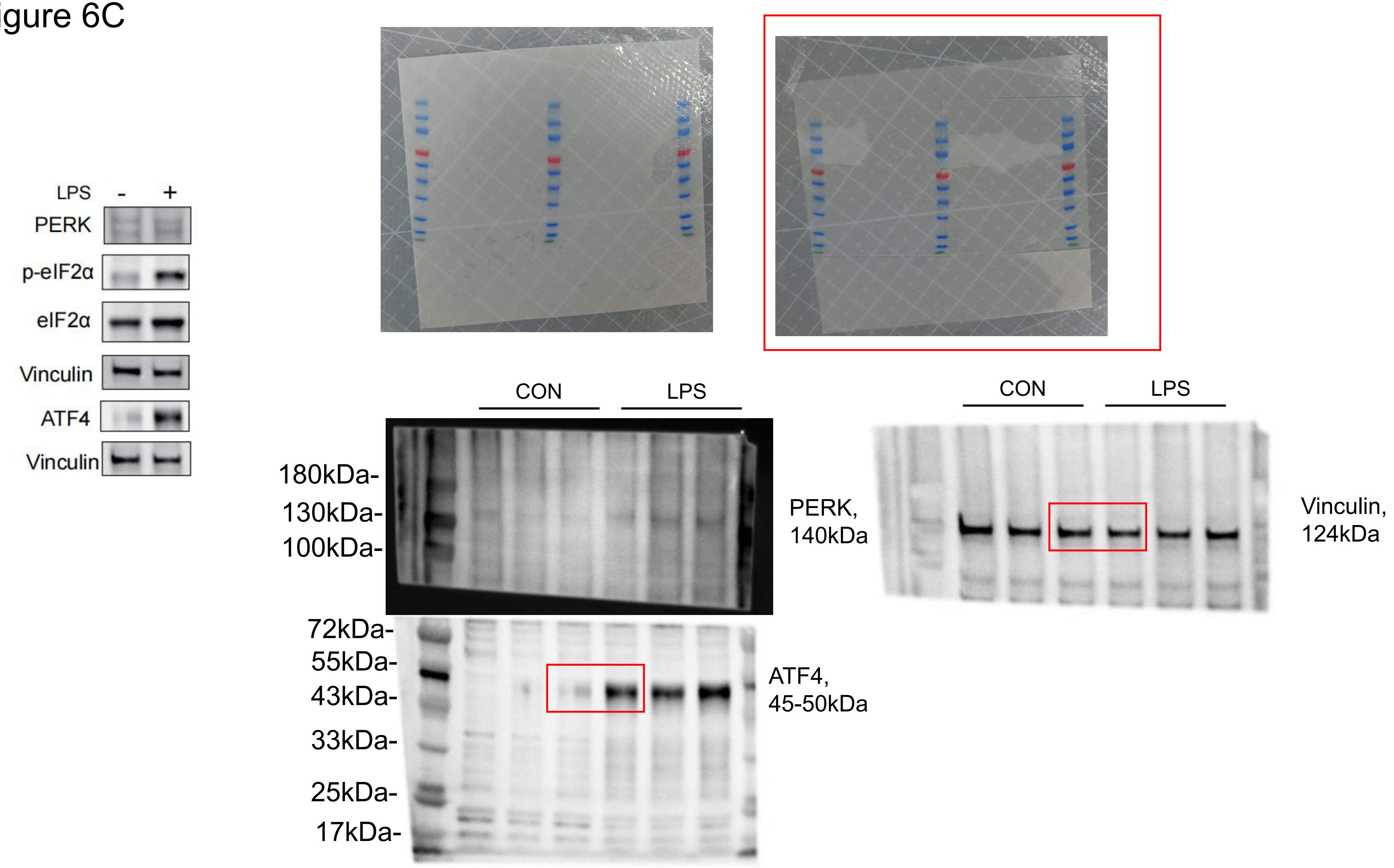

LPS - +
PERK
p-eIF2α
eIF2α
Vinculin
ATF4
Vinculin
CON
LPS
180kDa-
130kDa-
100kDa-
PERK,
140kDa
72kDa-
55kDa-
43kDa-
33kDa-
25kDa-
17kDa-
ATF4,
45-50kDa
CON
LPS
Vinculin,
124kDa

# Figure 6C

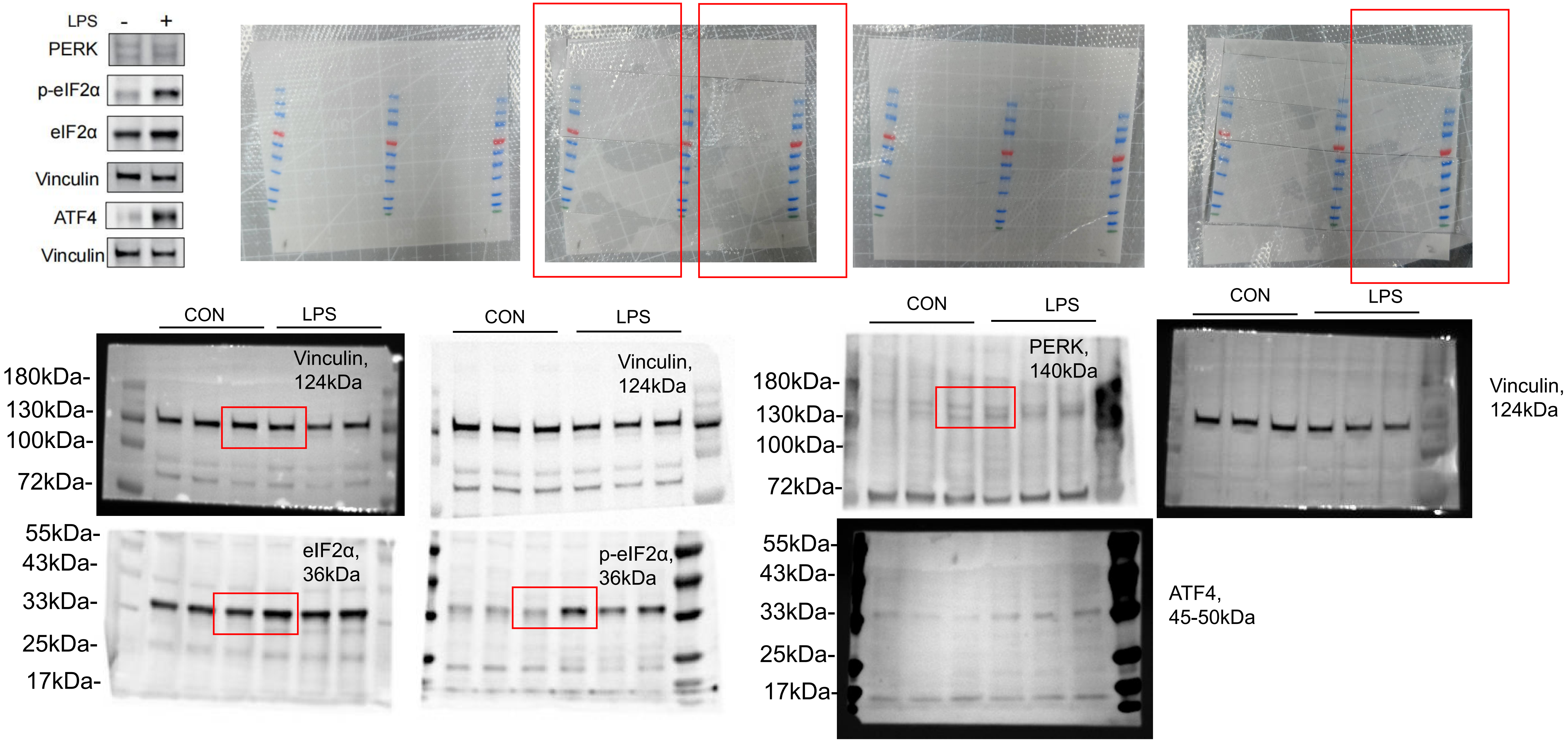
LPS - +
PERK
p-eIF2α
eIF2α
Vinculin
ATF4
Vinculin
CON
LPS
180kDa-
130kDa-
100kDa-
72kDa-
Vinculin, 124kDa
55kDa-
43kDa-
33kDa-
25kDa-
17kDa-
eIF2α, 36kDa
p-eIF2α, 36kDa
PERK, 140kDa
ATF4, 45-50kDa

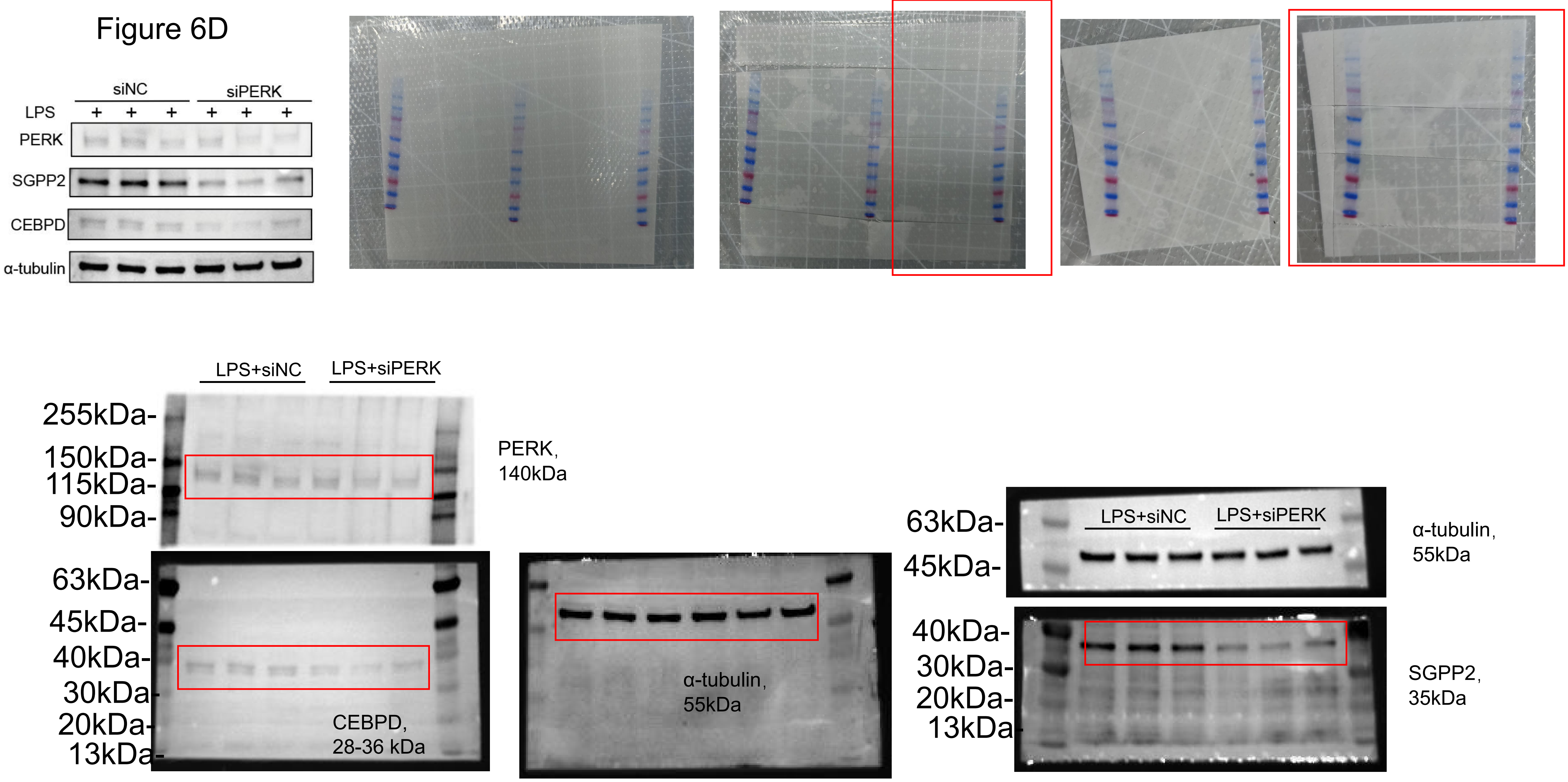
Figure 6D
siNC
siPERK
LPS
+ + + + + +
PERK
SGPP2
CEBPD
α-tubulin
LPS+siNC
LPS+siPERK
255kDa-
150kDa-
115kDa-
90kDa-
PERK,
140kDa
63kDa-
45kDa-
40kDa-
30kDa-
20kDa-
13kDa-
CEBPD,
28-36 kDa
α-tubulin,
55kDa
63kDa-
45kDa-
LPS+siNC
LPS+siPERK
α-tubulin,
55kDa
40kDa-
30kDa-
20kDa-
13kDa-
SGPP2,
35kDa

# Figure 6E

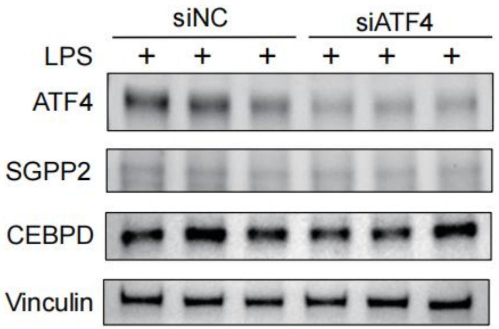
siNC
siATF4
LPS
+ + + + + +
ATF4
SGPP2
CEBPD
Vinculin

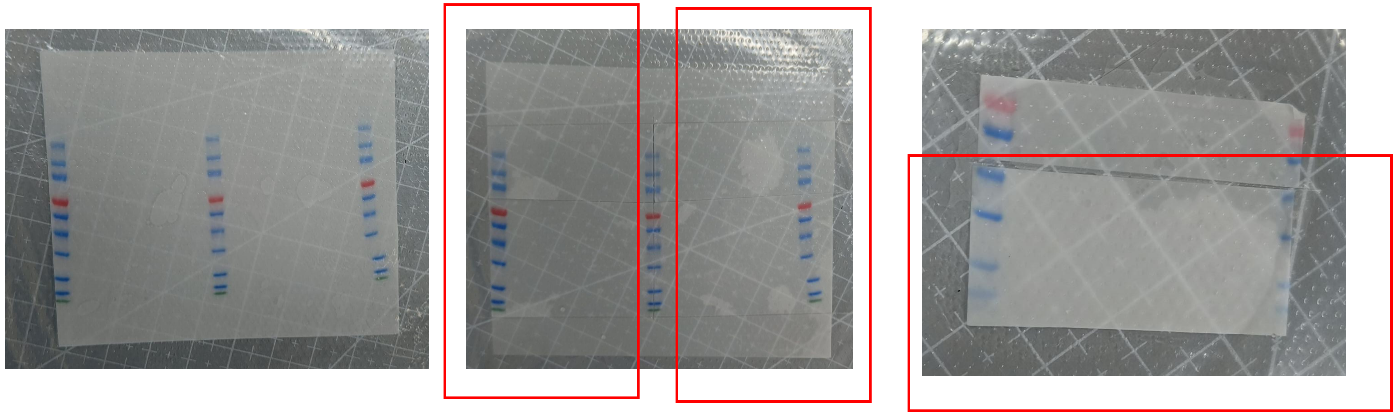

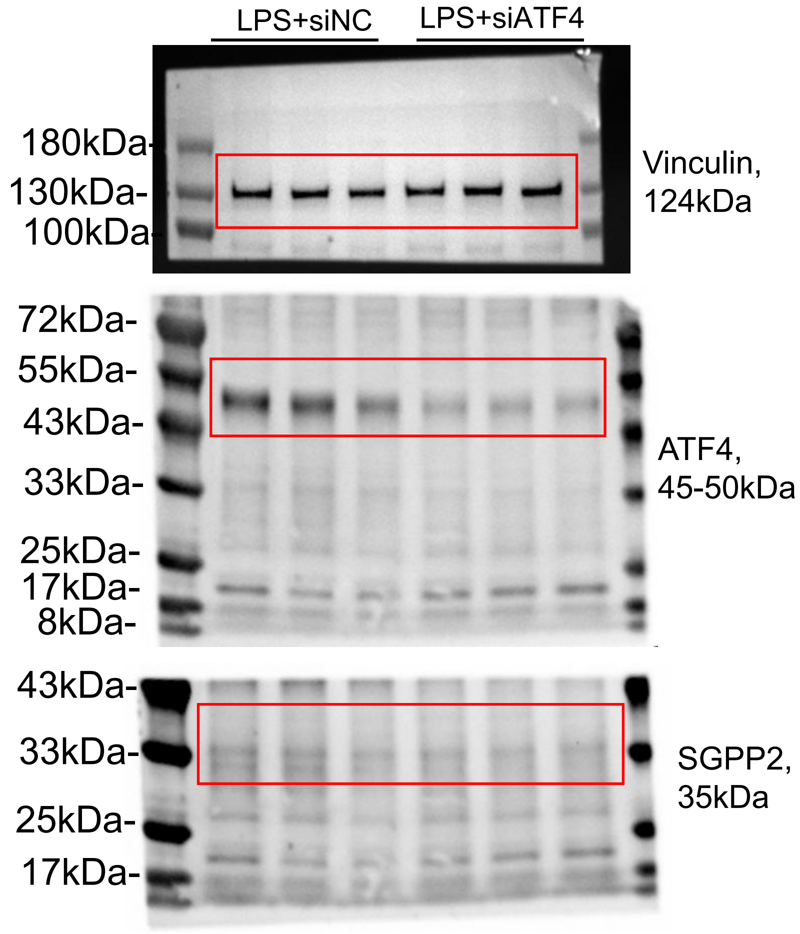
LPS+siNC
LPS+siATF4
180kDa-
130kDa-
100kDa-
Vinculin, 124kDa
72kDa-
55kDa-
43kDa-
33kDa-
25kDa-
17kDa-
8kDa-
ATF4, 45-50kDa
43kDa-
33kDa-
25kDa-
17kDa-
SGPP2, 35kDa

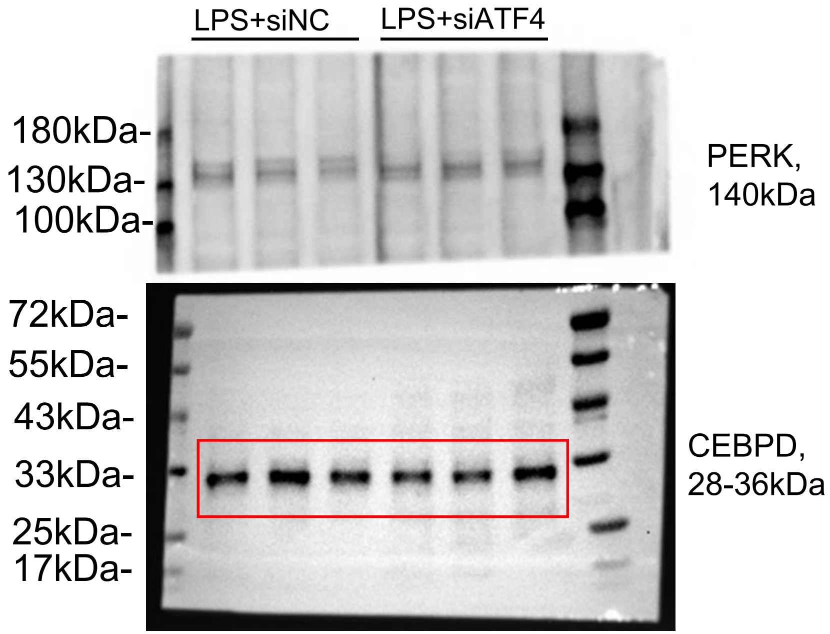
LPS+siNC
LPS+siATF4
180kDa-
130kDa-
100kDa-
PERK, 140kDa
72kDa-
55kDa-
43kDa-
33kDa-
25kDa-
17kDa-
CEBPD, 28-36kDa

Figure S2.A

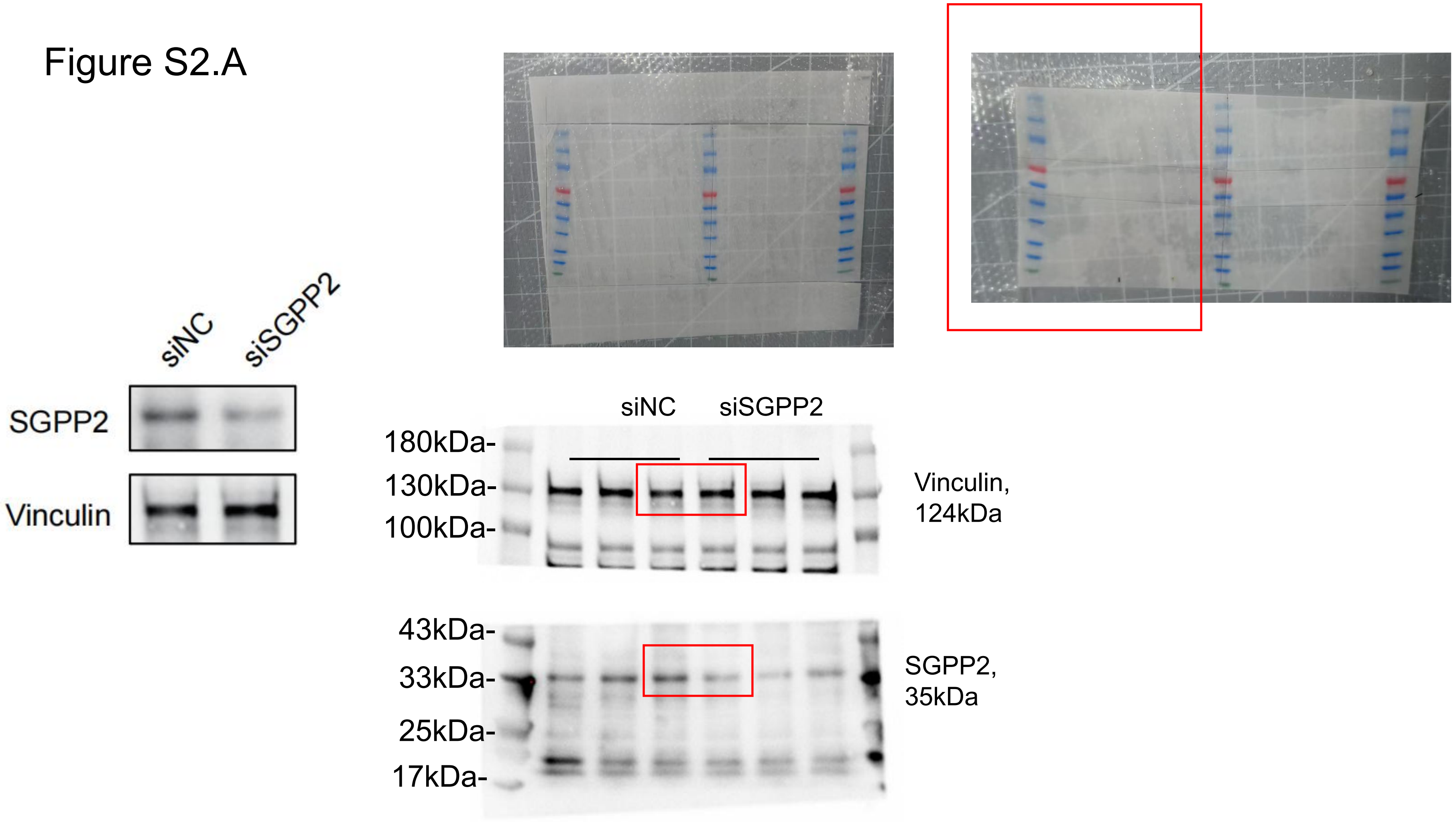

siNC
siSGPP2
SGPP2
Vinculin
siNC
siSGPP2
180kDa-
130kDa-
100kDa-
Vinculin, 124kDa
43kDa-
33kDa-
25kDa-
17kDa-
SGPP2, 35kDa

Figure S2.B

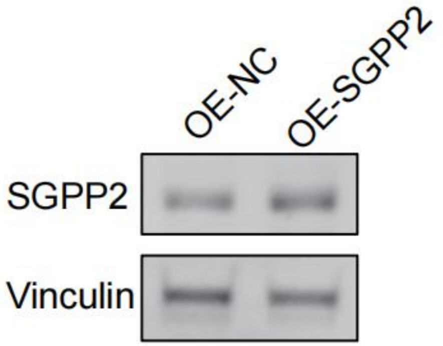
OE-NC
OE-SGPP2
SGPP2
Vinculin

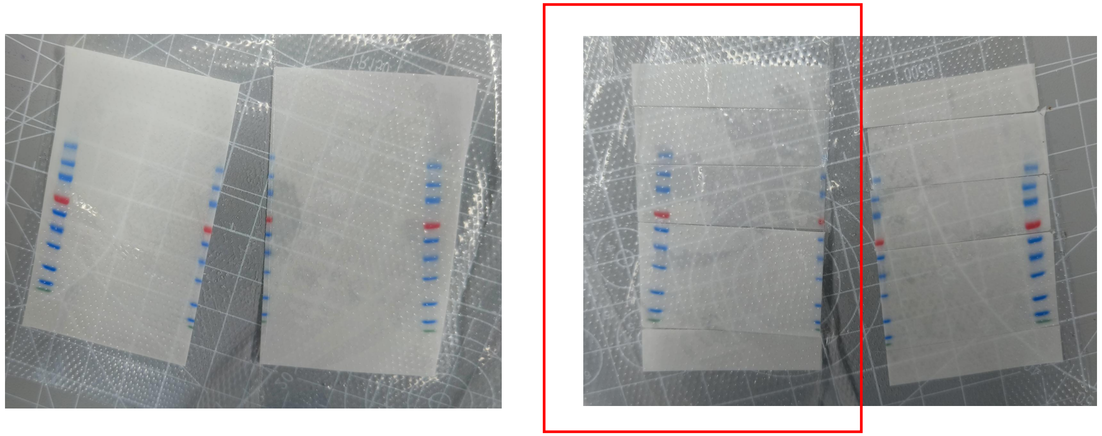

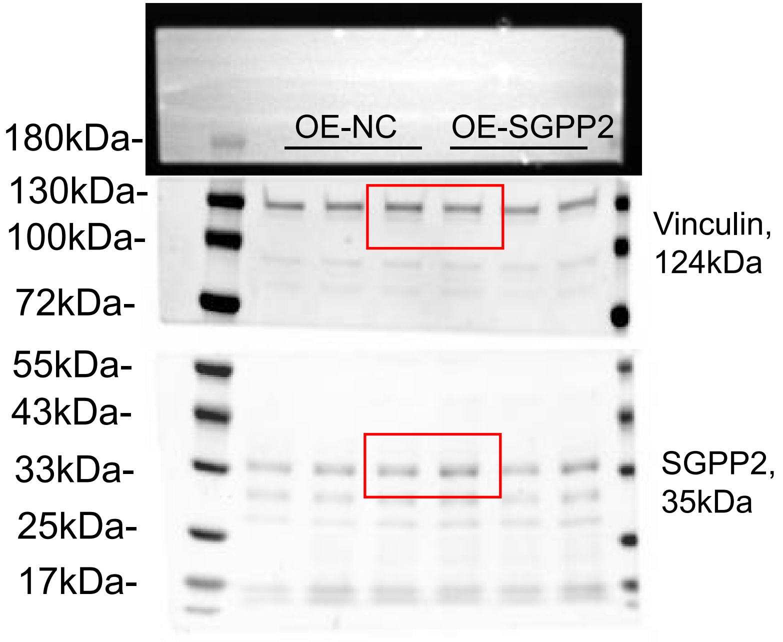
180kDa-
130kDa-
100kDa-
72kDa-
55kDa-
43kDa-
33kDa-
25kDa-
17kDa-
OE-NC
OE-SGPP2
Vinculin,
124kDa
SGPP2,
35kDa
